# Latitudinal and temporal distribution of aerosols and precipitable water vapor in the tropical Andes from AERONET, sounding, and MERRA-2 data

**DOI:** 10.1038/s41598-024-51247-9

**Published:** 2024-01-09

**Authors:** María Cazorla, David M. Giles, Edgar Herrera, Luis Suárez, Rene Estevan, Marcos Andrade, Álvaro Bastidas

**Affiliations:** 1 Universidad San Francisco de Quito USFQ, Instituto de Investigaciones Atmosféricas, Quito, Ecuador; 2 Science Systems and Applications, Inc. (SSAI), Lanham, MD USA; 3 NASA Goddard Space Flight Center (GSFC), Greenbelt, MD USA; 4 Instituto Geofísico del Perú, Huancayo, Peru; 5 Laboratorio de Física de la Atmósfera, Universidad Mayor de San Andrés, La Paz, Bolivia; 6 Department of Atmospheric and Oceanic Sciences, University of Maryland, College Park, MD USA; 7 Universidad Nacional de Colombia Sede Medellin, Medellin, Colombia

**Keywords:** Atmospheric chemistry, Atmospheric science, Environmental impact

## Abstract

The aerosol and precipitable water vapor (PW) distribution over the tropical Andes region is characterized using Aerosol Robotic Network (AERONET) observations at stations in Medellin (Colombia), Quito (Ecuador), Huancayo (Peru), and La Paz (Bolivia). AERONET aerosol optical depth (AOD) is interpreted using PM_2.5_ data when available. Columnar water vapor derived from ozone soundings at Quito is used to compare against AERONET PW. MERRA-2 data are used to complement analyses. Urban pollution and biomass burning smoke (BBS) dominate the regional aerosol composition. AOD and PM_2.5_ yearly cycles for coincident measurements correlate linearly at Medellin and Quito. The Andes cordillera’s orientation and elevation funnel or block BBS transport into valleys or highlands during the two fire seasons that systematically impact South America. The February–March season north of Colombia and the Colombian-Venezuelan border directly impacts Medellin. Possibly, the March aerosol signal over Quito has a long-range transport component. At Huancayo and La Paz, AOD increases in September due to the influence of BBS in the Amazon. AERONET PW and sounding data correlate linearly but a dry bias with respect to soundings was identified in AERONET. PW and rainfall progressively decrease from north to south due to increasing altitude. This regional diagnosis is an underlying basis to evaluate future changes in aerosol and PW given prevailing conditions of rapidly changing atmospheric composition.

## Introduction

**Figure 1:**
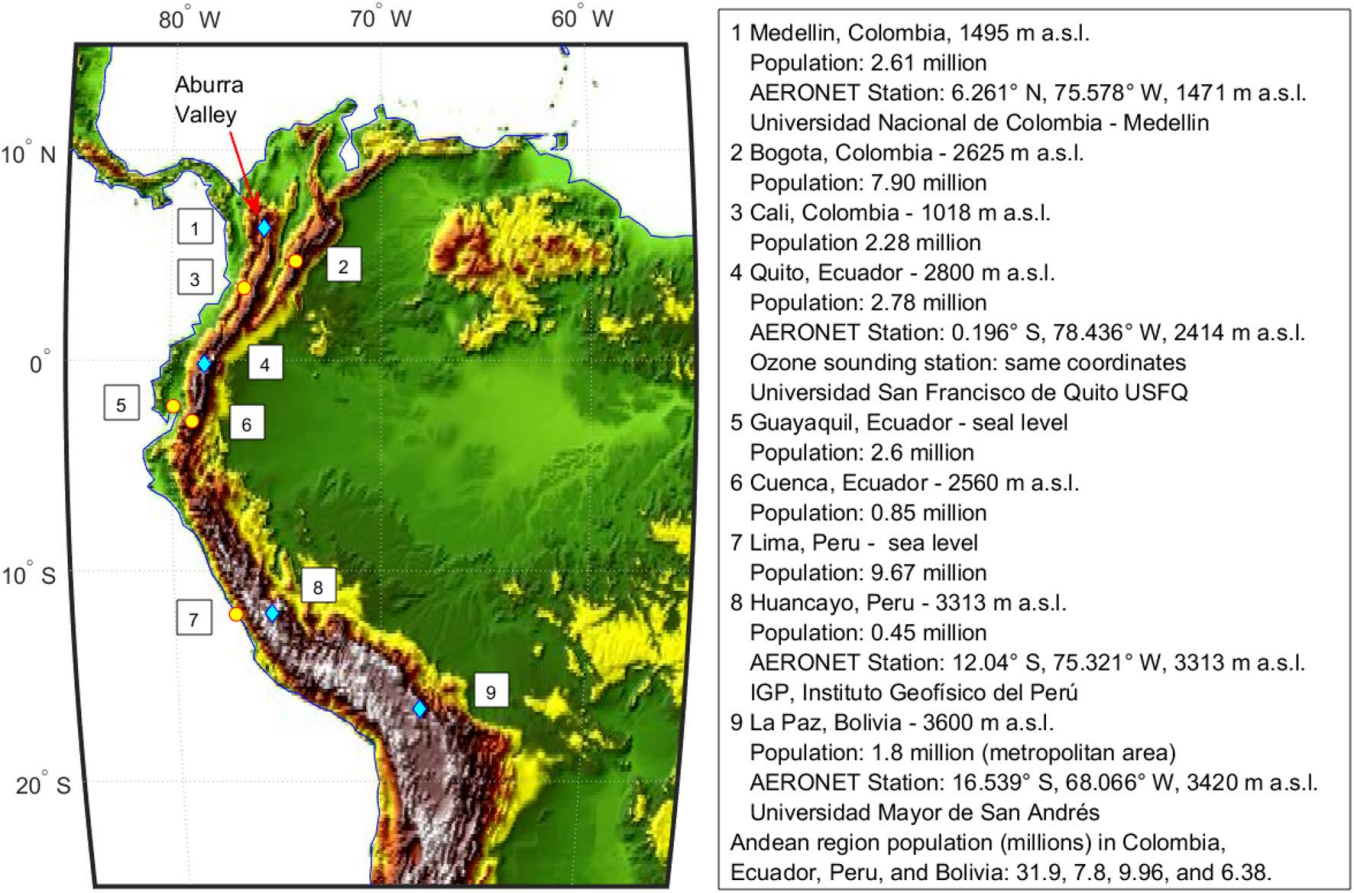
Topographic map of the tropical Andes. Yellow circles and numbers show the location of several cities in Andean countries. Cyan diamonds indicate cities that have AERONET stations. The red arrow indicates the location of the Aburra Valley. The legend to the right indicates cities’ altitude, population (from national statistics^1^), and coordinates of AERONET stations (V3, Level 2, multi-year data), where available. This map was created using MATLAB R2022b for Academic Use licensed to USFQ (https://la.mathworks.com/).

Understanding the current distribution of aerosols and precipitable water vapor (PW) over climate vulnerable regions such as the tropical Andes is critical given that atmospheric composition continues to change rapidly and globally. The tropical Andes mountains are densely populated, unlike other mountain regions in the world, a remarkable feature in addition to their high altitude and continental scale length^1^. Examples of large and medium-size Andean cities are Medellín, Bogotá, Cali, Quito, Cuenca, Huancayo, and La Paz, but numerous towns of varied sizes are distributed all along Andean valleys and highlands (Fig. 1). Thus, climate risks associated with changes in atmospheric aerosols and PW directly affect Andean populations. For example, Andean glaciers are melting more rapidly due to deposition of black and brown carbon and reducing freshwater reservoirs utilized in this region^2^. In addition, air pollution levels have elevated to the point of causing seasonal air quality emergencies in cities such as Bogota due to the transport of biomass burning smoke from Orinoco savanna fires mostly during the month of March^3^. However, a more extensive coordinated investigation on the characteristics and origin of atmospheric aerosols and how they become distributed along the tropical Andes has been lacking. A dedicated look at PW in connection to rainfall is also necessary given that climate projections indicate that extreme precipitation events are expected to increase in the northwestern South American region, while more southern Andean latitudes are expected to suffer increased droughts^1,4^. Here, we present a consolidated study that delves into the status and changes in near surface and columnar aerosols and water vapor over the tropical Andes, which is relevant at a time of imminent climate change.

Given the complexity of the terrain and the continental span of the Andes, an important angle to consider is how topography influences the distribution of aerosols and PW. The narrow width of the Andean cordillera abruptly divides South America and separates the Amazon from the Pacific Ocean. Consequently, urban canopies intermingle with cloud forests, and more arid regions. This yields an intricate combination of surface types and emissions across a large altitudinal gradient. Moreover, east of the Andes, the Amazon watershed is an important source of humidity, unfortunately also of biomass burning emissions. The Andes mountains act as a barrier to reduce biomass burning smoke transport from the Amazon to the high altitude La Paz region, although smoke intrusions that led to increased background levels of aerosols were observed during such a season^5^. North of the equator, biomass burning smoke transport directly affects cities in the Colombian Andes^6,7^. Over the Ecuadorian highlands, studies indicate that the Andes induce mechanical blocking as the signature of polluted air masses is generally absent in vertical profiles of tracers such as ozone^8,9^. However, the extent of this effect has not been investigated from the perspective of aerosol distribution. Previous studies suggest mixed influences of topography in connection to biomass burning smoke transport. Furthermore, the altitudinal gradient induces a mixture of arid and wet weather patterns, which produce conditions impacting the abundance of aerosols and PW in the atmospheric column. In the present study, we explore these effects from an observational perspective.

In this work we characterize the regional and seasonal distribution of aerosols based on AERONET and surface data. Furthermore, we use columnar water vapor measurements performed during regular ozone soundings launched from Quito (Ecuador) to compare against AERONET PW observations. To complement our analysis, we use Modern-Era Retrospective analysis for Research and Applications, Version 2 (MERRA-2) data to investigate the aerosol composition and PW distribution in the region. In the “Methods” section we describe in detail data sets and analysis techniques used in this study. The main findings are discussed in “Results and discussion” and summarized in the “Conclusions” section.

## Methods

### AERONET data sets

The regional distribution of aerosols and PW, their annual cycles, and aerosol characteristics in connection to the topographic effects that the Andes induce are studied between latitudes 6° N and 16.5° S using Version 3 Level 2.0 AERONET data^10,11^. Multi-year observations are provided from stations in Colombia, Ecuador, Peru, and Bolivia. Below we discuss technical aspects of data sets and study time periods.

The aerosol robotic network (AERONET) is a global federated network of surface-based remote sensing instruments that provide high quality measurements of aerosol optical depth (AOD) and retrievals of aerosol characteristics and water vapor^10,12,13^. The AERONET network deploys Cimel Electronique Sun-sky-Moon radiometers to perform direct Sun spectral measurements at 340, 380, 440, 500, 675, 870, 1020, and 1640 nm wavelengths, while sky scanning measurements use four wavelengths: 440, 675, 870, and 1020 nm. This study uses Version 3, Level 2.0 data that were accessed from the AERONET website (https://aeronet.gsfc.nasa.gov/). The AERONET site name, coordinates, and institutions hosting AERONET instruments in Andean countries of this study are depicted in Fig. 1. The AOD measurements have an uncertainty from 0.01 to 0.02 with the maximum uncertainty only at the UV wavelengths^10^. The Angström exponent (AE) is derived from AOD measurements between 440 and 870 nm and this parameter provides an index on the relative size of aerosol particles where values near 0.0 indicate large particles and values near 2.0 indicate small particles^14^.The total column precipitable water (PW) measurements are calculated by extrapolating the direct Sun measured transmission from 675 and 870 nm to 940 nm and subtracting the measured transmission at 940 nm, using absorption constants specific to the 940 nm filter, and water vapor optical air mass with an uncertainty of 10%^10,15^. The Spectral Deconvolution Algorithm (SDA) retrievals derived from spectral direct Sun AOD measurements provide the AOD fine-mode, coarse-mode, and fine mode fraction (FMF) at 500 nm^10,11,16,17^. Version 3, Level 2.0 aerosol volume size distributions and single scattering albedos (SSA) at 440, 675, 870, and 1020 nm were retrieved from spectral AOD and almucantar and hybrid sky scan radiance measurements^11,18^. The Andean sites were evaluated during the following periods: 2012–2020 (Medellin), 2017–2022 (Quito), 2015–2021 (Huancayo) and 2006–2022 (La Paz). Unavailable periods at some stations are possibly due to maintenance, calibration, or data quality issues. The sampling timeline and total observations available for each AERONET product and station are depicted in Fig. S1 and Table S1. In this work, AERONET time series are presented with the original temporal resolution (5 min), while annual cycles were obtained using all data to calculate monthly means.

### Sounding columnar water vapor measurements

Ozone soundings have been regularly launched since 2014 from the Atmospheric Measurement Station at Universidad San Francisco de Quito (EMA USFQ, Spanish acronyms) following data quality assurance protocols^8,9,19^. This is the same station and institution that hosts an AERONET instrument in Quito. During a sounding, P-T-U (pressure, temperature, humidity) are measured with InterMet Systems radiosondes (models iMet-1RSB from 2014 to 2019 and iMet-4RSB from 2019 until the present). In this study, we use 78 vertical profiles of water vapor mixing ratio collected between June 2014 and June 2022. These sounding data are available at: https://observaciones-iia.usfq.edu.ec/, under ‘USFQ Data\Sondeos\EMA_Soundings\’. The water vapor mixing ratio is integrated from the surface up to 17 km a.s.l. (average depth of the troposphere at this location^8,19^) to determine the water vapor column. Uncertainty in iMet humidity measurements is 5%^20^.

Sounding data are presented in time series along with AERONET and MERRA-2 PW. A 1:1 comparison between AERONET PW data (5-min) and sounding measurements was performed by interpolating AERONET data to the soundings time stamp. A total of 49 data pairs were compared during the period. The number of data points available per time window (period within which both measurements are available) is the following: 17 points within 0–0.5 h, 2 points within 0.5–1 h, 6 points within 1–2 h, 22 points within 2–24 h, and 2 points within 24–49 h. In the linear regression, points were colored by time window to detect possible differences due to time lapse between AERONET and soundings but no evidence of data clustering according to time window was found. As a check, AERONET PW daily averages were compared to soundings performed on the same day with essentially the same results. A 1:1 comparison was also performed between 1-h MERRA-2 Total precipitable water vapor (described below) and sounding columnar measurements. To this end, MERRA-2 data were interpolated to the soundings time stamp. MERRA-2 data were available within 1 h of sounding launch times. A total of 78 data pairs were compared.

### PM_2.5_ and humidity observations

PM_2.5_ observations were used to interpret whether the AERONET fine-mode AOD columnar measurement at city stations is due to urban aerosols. These measurements were also interpreted with the help of specific humidity calculated from surface data (pressure, temperature, and relative humidity).

For the Medellin region, PM_2.5_ and meteorological data are publicly available from the Aburra Valley Early Warning System (SIATA, Spanish acronym, https://siata.gov.co/siata_nuevo). Universidad Nacional de Colombia – Medellin (UNAL), the institution that hosts AERONET, is a site listed within SIATA. However, at this station only meteorological data are available, not PM_2.5_. Therefore, 1-h measurements of PM_2.5_ were downloaded from a station (Tráfico Centro) located 1.3 km away from UNAL. Data were acquired for the same period covered by AERONET. According to network documentation, PM_2.5_ is measured with a MetOne BAM 1020 instrument (additive bias + /– 2 µg m^–3^), and analytical methods meet US EPA standards^21^. In addition, meteorological data (1-min) were downloaded from the same website (UNAL station). Water vapor mixing ratio (q) was calculated using surface temperature, humidity, and pressure. Annual cycles of PM_2.5_ and q were prepared by overlapping all data in one year and extracting monthly means. In the case of PM_2.5_, only data coincident with AERONET measurements (1-h window) were used for comparisons against AERONET fine-mode AOD. Precipitation data available at the UNAL station covered 2013–2022.

For Quito, 1-h PM_2.5_ and meteorological data measured at a station located 4 km away from EMA USFQ were downloaded in 2022 from the Quito’s Secretariat for the Environment website (Tumbaco station) (https://ambiente.quito.gob.ec/). While this data set used to be publicly available for download, currently historical data needs to be requested to the Quito’s Secretariat for the Environment. Real time air quality index and 1-h averages in the current month are available at http://aireambiente.quito.gob.ec/. Thus, data used in this study are presented in Table S2. Thermo 5014i sensors (usually 5% uncertainty) are the type of instrumentation used by the Quito Air Quality Monitoring network to measure PM_2.5_^22^. To obtain PM_2.5_ and q annual cycles, data were treated in a similar manner as for the Medellin time series and for the period that overlaps with AERONET. Precipitation data (1-month) were obtained from 2006 to 2021.

At Huancayo, continuous PM_2.5_ monitoring is unavailable. Thus, we used measurements from a field campaign in March to November 2017 by Huamán De La Cruz et al.^23^. Precipitation data were obtained from a climatology (1991–2020) for Central Peru (Huayao station) prepared by IGP (Spanish for Peruvian Geophysical Institute, Table S3)^24^.

Finally, monthly averages of PM_2.5_ at La Paz (2021–2022) were extracted from Air Quality Reports by the local government^25,26^. According to this documentation, PM_2.5_ is measured by the MoniCA LP (Spanish acronym for Air Quality Monitoring at La Paz) network with a Beta attenuation instrument (usually 5% uncertainty) that complies with the Bolivian air quality legislation. Monthly precipitation data (2006–2022) were obtained from the National Statistics Institute website (INE, https://www.ine.gob.bo/index.php/medio-ambiente/clima-y-atmosfera/).

### Reanalysis data

The Modern-Era Retrospective analysis for Research and Applications, Version 2 (MERRA-2) is a NASA project enabling enhanced meteorological assimilation as well as incorporation of modern hyperspectral and microwave, GPS radio-occultation, and atmospheric composition observations^27^. The MERRA-2 Level 2 data were acquired from the Giovanni NASA website (https://giovanni.gsfc.nasa.gov/giovanni/). Total extinction aerosol optical thickness (AOT) 550 nm and contributions by black carbon, organic carbon, SO_4_, dust, and sea salt extinction AOT 550 nm were downloaded as monthly averages (2006–2022) for grid cells over the coordinates of each station^28^. This period (2006–2022) was chosen for expanded analysis for being equal to the longest AERONET time series in the region. For water vapor comparisons, 1-h MERRA-2 total precipitable water vapor data^29^ from 2014 to 2022 were obtained for a grid cell over the Quito coordinates to compared against sounding columnar water vapor. 1-month averages of MERRA-2 total precipitable water vapor were downloaded for the 2006–2022 period^30^ for the coordinates of each station. To summarize regional findings about aerosol and PW distribution, MERRA-2 maps were prepared from the Giovanni NASA website. The domain used was 82° W, 23° S, 55° W, 13° N. AOT 550 nm maps were obtained as recurring averages for March and September months over 10-years (2012–2022). Likewise, MERRA-2 PW maps for the same domain were obtained as time-average maps (2012–2022).

### HYSPLIT back trajectory model

The NOAA Hybrid Single-Particle Lagrangian Integrated Trajectory (HYSPLIT) model was used to compute archive backward trajectories on the READY website (https://www.ready.noaa.gov/HYSPLIT.php)^31,32^. The HYSPLIT back trajectory analyses were used to assess specific biomass burning smoke transport events based on the highest AOD measurements recorded in March 2020 at Medellin and Quito AERONET stations. The 96-h back trajectory analysis at three heights (500, 1500, and 3000 m a.g.l.) were generated from the Medellin station coordinates and ending on 26 March 2020 at 1300 UTC. From the Quito coordinates, back trajectory analysis ending on 25 March 2020 at 1300 UTC were run for 72 h at heights 1500, 2000, and 3000 m a.g.l. The total run time is such that the model starts on 23 March, a day when skies were clear and satellite images are of good quality. The two first height levels chosen for the Quito back trajectories are higher because this is a high-altitude location secluded by the Andean mountain range.

### Satellite images

MODIS (Terra and Aqua) and VIIRS true color imagery of northwestern South America was obtained from NASA Worldview (https://worldview.earthdata.nasa.gov) to illustrate case studies of biomass burning smoke transport in March 2020. Links to specific images are included in the Supplementary Information.

## Results and discussion

### Aerosol distribution

**Figure 2:**
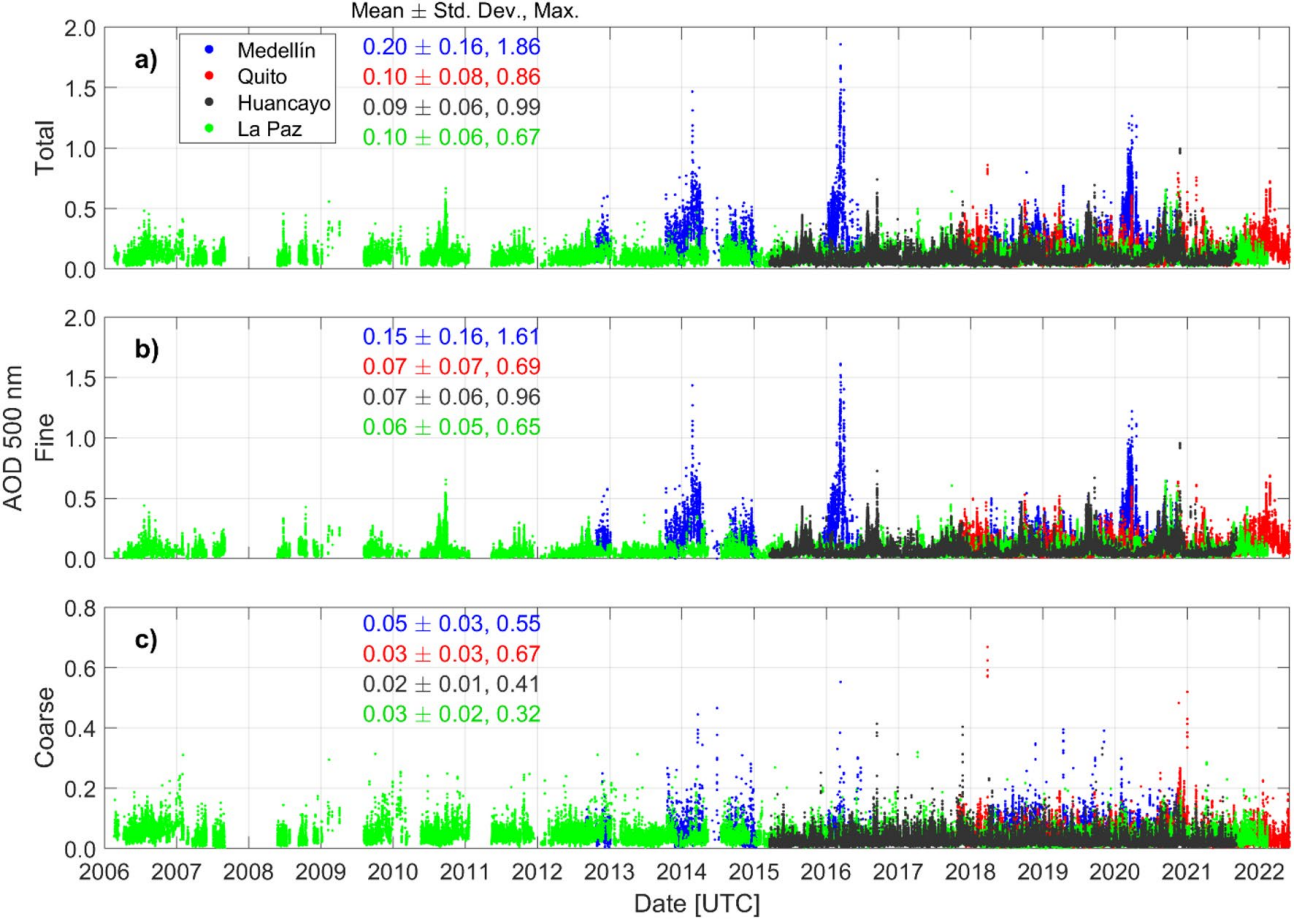
Multi-year Version 3, Level 2.0 AERONET Spectral Deconvolution Algorithm (SDA)^10,11^ time series of (**a**) Total, (**b**) Fine-mode, and (**c**) Coarse-mode AOD at 500 nm (5-min data) at stations Medellin (blue), Quito (red), Huancayo (black), and La Paz (green). The corresponding statistics by color indicate the time series mean with + /– one standard deviation, followed by the maximum value.

The aerosol optical depth (AOD) is the geophysical variable representing total column scattering and absorption (i.e., extinction) of the sunlight by aerosols. The AOD is proportional to the concentration where low values of the AOD indicate low aerosol concentrations in the total column along the measurement slant path to the Sun in which these measurements are translated to the vertical column by correcting for the optical air mass (or approximately 1.0/cos (solar zenith angle))^10^. The AOD can be computed for various wavelengths and typically referenced to the 500 nm maximum in solar irradiance and correspondence to many aerosol monitoring satellite retrieval bands for validation purposes^10^. The spectral direct sun measurements of AOD are used to retrieve the fine and coarse mode separation of the AOD using the Spectral Deconvolution Algorithm (SDA) retrieval^16,17^. The SDA AOD (500 nm) measured at Medellín, Quito, Huancayo, and La Paz are depicted in Fig. 2. La Paz has the longest time series (epoch 2006), while the newest station is Quito (epoch October 2017). While there are nonuniform overlapping periods and the distance between stations is large, these observations make evident the long-term and episodic distribution of aerosol over this mountainous region. Latitudinally, a first feature to notice is the magnitude of total AOD 500 nm at Medellin (6.26° N) in comparison to Quito (0.196° S), and more southern stations Huancayo (12.04° S) and La Paz (16.54° S). Total and fine-mode AOD 500 nm at Medellin (5-min data) reach values above 1.6, while maxima at the rest of the stations remain below 1.0 (Fig. 2). Consequently, the mean total and fine-mode AOD 500 nm at Medellín (0.2 and 0.15) are twice the values at the rest of the stations (statistics in Fig. 2). The temporal distribution of AOD can be observed as annual cycles when data are overlapped in a year (Fig. S2). At Medellin, total and fine-mode AOD 500 nm are maximum around 0.5 in March (Fig. S2a,b), on average. At the equatorial Quito station, AOD 500 nm reaches maxima in February–March and in November near values of 0.18 (total) and 0.14 (fine-mode). Meanwhile, at stations Huancayo and La Paz, aerosols increase in July to November (Fig. S2g,h,j,k) around an AOD 500 nm of 0.14 (total) and 0.12 (fine-mode). The July-November biomass burning season in the Brazilian Amazon and its impact on adjacent areas is well documented^33–35^. Less known is the biomass burning season in northern South America, over the Colombian and Venezuelan grasslands, which peaks in February-March^3,6,36^. Thus, looking at the regional aerosol distribution in this study, we found that AERONET stations from the equator to the north share partial similarities in AOD seasonality during the first trimester (first 3 months of the year), while there are similarities between the two more southerly stations during the third and fourth trimesters. Below, we discuss local and regional processes that lead to these temporal and latitudinal features.

**Figure 3:**
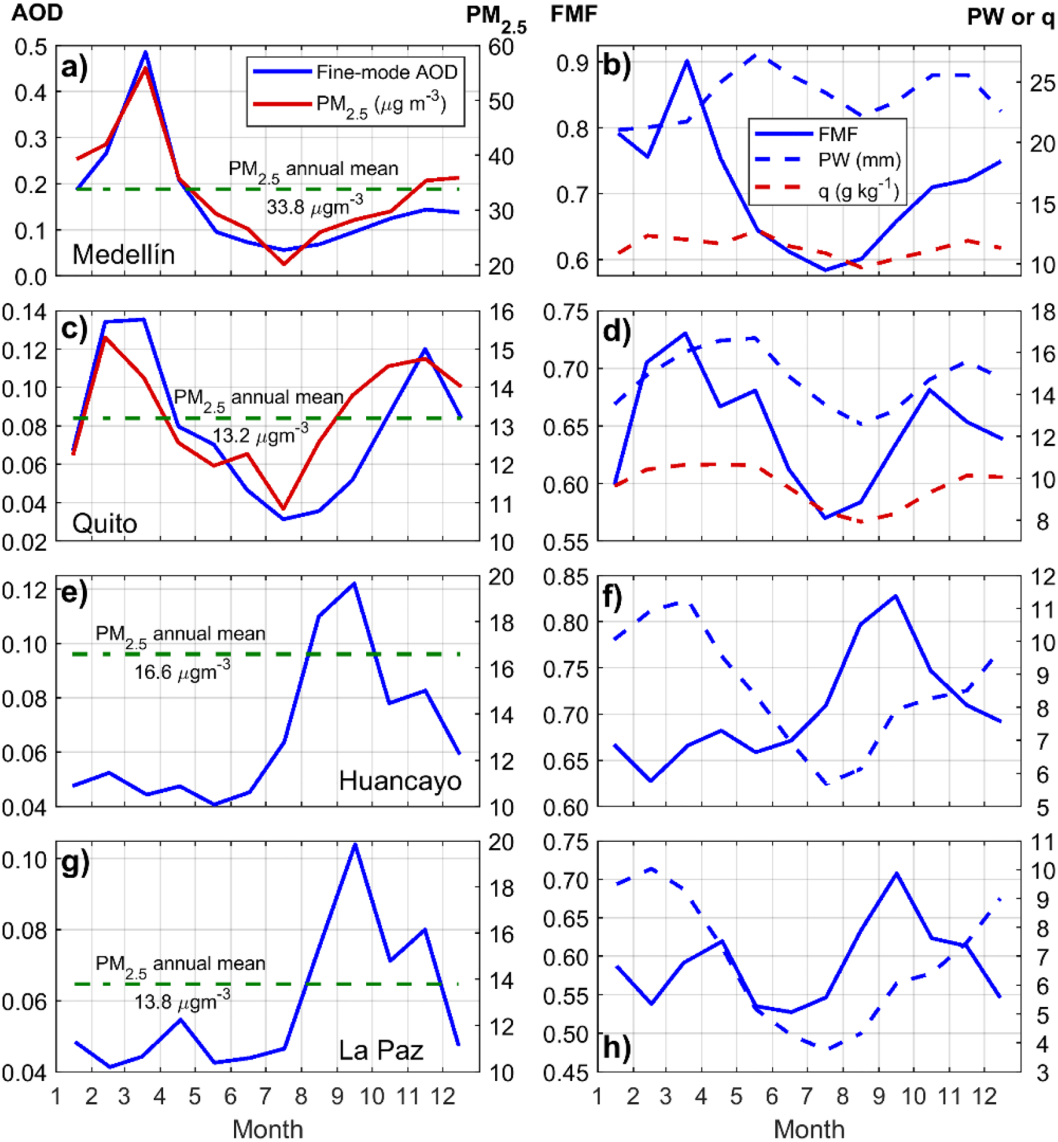
Left panels: AERONET Version 3 Level 2.0 SDA fine-mode AOD annual cycle (blue) together with PM_2.5_ (solid red) for coincident periods, where available. The mean of PM_2.5_ measurements is indicated with a green dashed line. Right panels: annual cycles of AERONET FMF (solid blue), PW (dashed blue), and water vapor mixing ratio (q, dashed red), where available. From top to bottom: (**a**) and (**b**) Medellin, (**c**) and (**d**) Quito, (**e**) and (**f**) Huancayo, (**g**) and (**h**) La Paz. PM_2.5_ and q were obtained from local air quality monitoring networks (Quito and Medellin) and from air quality reports and publications (Huancayo and La Paz), more in “Methods”.

Previous research by Casallas et al.^37^ characterized in detail PM_2.5_ in northwestern South America. They identified a distinct and high PM_2.5_ peak that seasonally develops in Medellin in February–March due to the transport of biomass burning smoke from wildfires in northern Colombia and the Colombian-Venezuelan border. Hence, Fig. 3a shows that the AERONET fine-mode AOD annual cycle peaks in March (also the FMF, Fig. 3b) and coincides with PM_2.5_ measured during the same period by the Medellin public network. Figure S3a shows the correlation between both annual cycles (R^2^ = 0.92). Thus, a maximum columnar value of 0.49 of fine-mode AOD contains an aerosol signal within the boundary layer of 56 µg m^–3^ of PM_2.5_, on average. An important aspect that plays a role in the development of pollution events at Medellin is that it is situated within the Aburra valley, where mountains surrounding the area make it difficult for local and transported contaminants to efficiently undergo dispersion^38,39^. The work by Casallas et al.^37^ suggests that urban PM_2.5_ pollution from traffic has been mitigated in Medellin by implementing electric and hybrid public transportation in lieu of former fossil fuel-based transportation. Outside February and March, the rest of the year PM_2.5_ is typically lower. Correspondingly, AERONET observations show that in months different from the biomass burning season, the fine-mode AOD remains below 0.2, on average.

In the case of Quito, the fine-mode AOD annual cycle can be interpreted with respect to the PM_2.5_ annual cycle calculated from 1-h data collected at a neighboring station (Methods). Figure 3c shows agreement between the times of the year when PM_2.5_ and AOD reach maxima Fig. S3b shows the correlation between both annual cycles (R^2^ = 0.63). Consistently, the AOD FMF peaks in the same months (Fig. 3d). From local knowledge and previous research, sources of pollution in the Quito region are typically of an urban nature, traffic, and industry emissions^22^. The school year begins in mid-September, for which traffic increases substantially and leads to higher PM_2.5_ until it slowly subsides during the months of summer vacation (July to August). In the outskirts of the city, to the north and east, there are quarry activities (for construction materials such as limestone, gravel, and pumice) that are known to be sources of coarse mode aerosol particles^22^. Wildfires in the surrounding woods occur episodically at the end of the summer months during dry years (for example, events in September 2015, 2017, and 2018^40^). Aside from these more local episodes around Quito, the notion of a well-established biomass burning season occurring at an upwind location has not been identified as a systematic source of particles that affect air quality, unlike at Medellin and Bogotá. However, AERONET columnar measurements are capable of detecting signals above the boundary layer in the free troposphere. With Quito located to the south-southwest of the Colombian and Venezuelan regions where biomass burning is a seasonal occurrence, the question arises on whether Quito is affected by long-range transport of polluted air masses whose signals could be detected by AERONET. In the following section we explore this hypothesis. Another factor to consider is the onset of the rainy seasons, which in Quito – and in the rest of the Ecuadorian highlands – occur in March to May and around November. The first season is due to the ITCZ oscillating around the equator near March^41^, while the second season is more convective in nature that follows the warmer summer months combined with humid air masses transported from the Amazon (a more complete description of weather patterns at the Quito station can be found elsewhere^42^). Hence, the annual cycles of PW and q coincide (Fig. 3d). Former research showed that the boundary layer is less deep in March and November at Quito^42^, for which a shallow mixing volume plays a role in inducing higher PM_2.5_ concentrations near the surface. Although precipitation has the effect of cleaning the atmosphere from contaminants through wet deposition, March and November are also the months when the water vapor content in the air is the highest in the Quito region. Previous research demonstrated the relationship between humidity and growth of sulfate and nitrate aerosols, and to a lesser extent of biomass burning smoke^43^. At Quito, humidity possibly plays a role in the development of increased aerosol concentrations near the surface around March and October–November due to increased precursors from traffic followed by hygroscopic particle growth. In further sections, we explore this influence from the perspective of aerosol contributions to the total loading, particularly with respect to sulfate particles.

Unlike the northern Andes locations, Huancayo and La Paz fine-mode AOD 500 nm values are higher from August to November (Fig. 3e and g). During this season at Huancayo, 5-min measurements mostly reached 0.7 except for one event on 24 November 2020 when six measurements ranged 0.9–0.96 due to enhanced fire activity nearby (Fig. S4). Previous work showed that the biomass burning season at the Amazon impacts the atmospheric aerosol loading over the Peruvian Andes, which is maximized in September^44^. Hence the FMF annual cycle also peaks in September (Fig. 3f). Continuous PM_2.5_ monitoring at Huancayo is unavailable for which finding temporal features in the annual cycle to relate to AERONET measurements is currently not possible. A 2017 measurement campaign at metropolitan Huancayo (March to November) quantified PM_2.5_ concentrations and characterized particles chemically^23^. The mean concentration of PM_2.5_ during the campaign was 16.6 µg m^–3^ and the maximum 36.8 µg m^–3^. Higher concentrations were observed during the dry season (May to September), and it was determined that variability exists among sampling sites. Due to the high detected levels of PM_2.5_ at metropolitan Huancayo, the authors of the study concluded that a plan to improve air quality is necessary. Although Huancayo might be regarded by some as a background atmosphere station, we find that some regions at Huancayo could reach mean surface levels of PM_2.5_ comparable to the mean at Medellin (33.8 µg m^–3^), and concentrations are higher than the mean at Quito (13.2 µg m^–3^). However, the lack of continuous measurements limits more quantitative and seasonal assessments. Among the soluble ions found in PM_2.5_ samples, nitrate, ammonia, and sulfate fractions were reported and attributed to traffic and farming activities in the area. This latter aspect is relevant to the present study with respect to findings about aerosol properties and composition that we explain further below.

At La Paz, the fine-mode AOD 500 nm and FMF annual cycles have the same temporal features as the ones at Huancayo (Fig. 3g and h). These findings agree with research that investigated extensively the La Paz AERONET time series until 2014 and compared against AERONET observations at the Bolivian lowlands and the adjacent Brazilian Amazon^5^. Thus, it was found that during the biomass burning season, while maxima for daily averages of the fine-mode AOD 500 nm at La Paz were about 0.4, at the lowland stations (i.e. Santa Cruz, Bolivia) maxima neared 2.0^5^. Hence, it was demonstrated that the Andes mountains curb the bulk of the smoke advection from the Amazon as the low-level jet, that runs parallel to the cordillera from north to south, diverts air masses in the subtropical direction^45^. In the present work, the updated annual cycle of the fine-mode AOD 500 nm at La Paz (2006–2022 data, Fig. 3g) shows that an otherwise clean baseline (0.05) doubles in the month of September. As at Huancayo, La Paz PM_2.5_ data is not available from a public network. However, from air quality reports by the Bolivian environmental authority^25,26^ the PM_2.5_ annual mean (2021 and 2022) was 13.8 µg m^–3^, which relates to the low baseline fine-mode AOD during most of the year.

**Figure 4:**
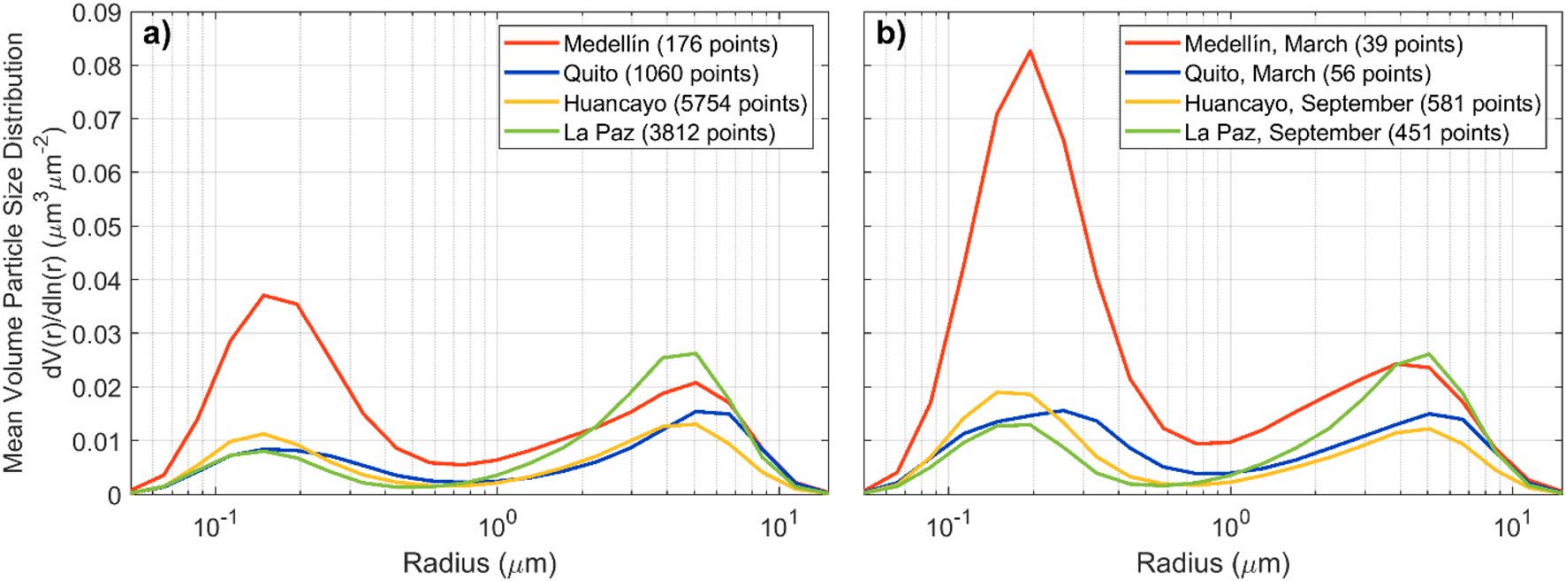
Mean volume particle size distribution from available AERONET Version 3 Level 2.0 hybrid and almucantar retrievals at Medellin (red), Quito (blue), Huancayo (yellow), and La Paz (green) for (**a**) all points, (**b**) only peak months during biomass burning seasons (March at Medellin and Quito, September at Huancayo and La Paz).

In addition to the information that local PM_2.5_ measurements provide, the contributions of the fine and coarse aerosol modes to the total columnar AERONET measurements can be assessed through the mean volume particle size distribution from hybrid and almucantar retrievals (Fig. 4). The magnitude of the columnar size distributions is generally low, partially due to limitations in the number of retrievals (mainly at Medellin and Quito during biomass burning months, Fig. 4b), which demand clear skies, high AOD loading (> 0.4) at large solar zenith angles (> 50° for almucantars and > 25° and < 75° for hybrids)^11^. From a qualitative perspective, however, the relative magnitude of the fine mode to the coarse mode distributions help reveal the aerosol columnar composition and differences among stations. For example, at Medellin the fine aerosol mode generally dominates, magnifies in March, and is substantially larger than the fine mode at the rest of the stations. These observations directly relate to findings about biomass burning smoke transport discussed earlier. At Quito, Huancayo, and to a lesser extent at La Paz, the fine aerosol mode increases during the peak months of the biomass burning influence (Fig. 4b) when compared to the bulk of the data (Fig. 4a).

As presented, urban aerosols in the densely populated Andean tropics become mixed with biomass burning smoke and have varying impacts by region and season. The complex Andean topography either channels or blocks pollution advection. At the northern Medellin location, smoke aerosols fill the Aburra valley during the first trimester, as the orientation of the three branches of the Andes cordillera lay parallel to the region where biomass burning occurs. Meanwhile, the equatorial Quito station, being more secluded within the Andean highlands, is not directly impacted, although it is possible that some of the AOD signal in March is partially due to aerosol long-range transport, which we explore further in the following section. Meanwhile, Huancayo and La Paz lay adjacent to the Amazon that typically burns extensively at the end of the dry season from August to October. The Andes effectively block transport of the bulk of biomass burning smoke, but some transport does raise aerosol background levels.

### Transport of biomass burning smoke case studies

**Figure 5:**
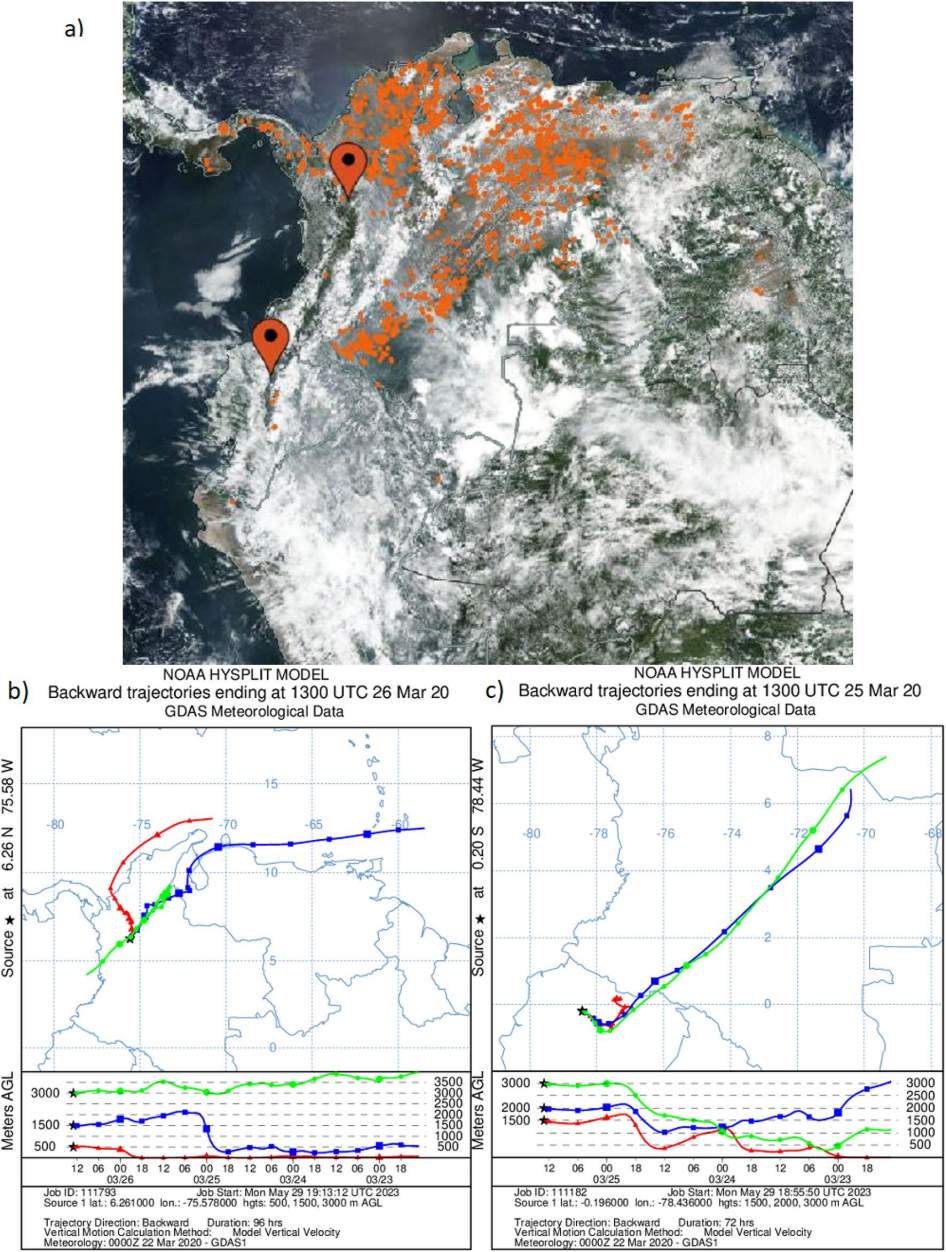
(**a**) MODIS (Terra and Aqua) and VIIRS true color image of Northwestern South America with thermal anomalies (in red) indicating fire locations on 23 March 2020. (**b**) HYSPLIT backtrajectory (GDAS meteorological data) run from Medellin coordinates and ending on the 26th of March of 2020. (**c**) Same but for the Quito coordinates and ending on the 25th of March 2020. The link to the MODIS image is: https://worldview.earthdata.nasa.gov/?v=-102.62785818145127,-13.872118489241101,-42.376425682402186,15.3121691274858&l=Reference_Labels_15m(hidden),Reference_Features_15m,Coastlines_15m,MODIS_Combined_Thermal_Anomalies_All,VIIRS_NOAA20_CorrectedReflectance_TrueColor(hidden),VIIRS_SNPP_CorrectedReflectance_TrueColor&lg=true&s=-78.4356,-0.1962%2B-75.578,6.2607&t=2020-03-23-T13%3A12%3A43Z.

To illustrate how the Medellin region is affected by smoke advection during the biomass burning season in northern Colombia and the border with Venezuela, an illustrative example is the high AOD event recorded in March 2020 (Fig. 2a). This case is relevant for analysis purposes because cities were under COVID-19 lockdowns, which mostly suppressed the aerosol urban signal. In Medellin and Bogota, the strict lockdown began on 20 March, which led to a decrease in primary pollutants, but levels of PM_2.5_ were high due to advection of biomass burning smoke^46^. Figure 5a depicts true color images from MODIS (Terra and Aqua) and VIIRS on the 23rd of March. Abundant fires are visible north and east of the Colombian Andes. Consistently, a HYSPLIT back trajectory analysis ending on the 26th of March, when total AOD was the highest (1.26 at 7:46 local time), shows that air masses within the boundary layer originated from burning areas at the north of Colombia and the northeast, over the border with Venezuela (Fig. 5b). During this event, PM_2.5_ was 68 µg m^–3^. At midnight the previous day the concentration was 90 µg m^–3^ (1-h data from Medellin public records, plot not shown).

As in the case of Medellin, March 2020 is a period when the potential impact of biomass burning smoke transport to the Quito region can be examined without the signal of urban aerosols. Previous research shows that during this period the ambient air concentrations of most pollutants, including PM_2.5_, dropped substantially^47,48^. However, on the 25th of March the AERONET fine-mode AOD 500 nm signal increased up to almost 0.6 as depicted in Fig. S5. At the same time, PM_2.5_ peaked at almost 40 µg m^–3^ (1-h mean), an unexpected value under no traffic conditions. In the absence of urban pollution, which could mask contributions from other sources in the AERONET measurement, these coincidental observations suggest that both signals are related. To better understand the origin of air masses during this event, a HYSPLIT back trajectory analysis ending on the 25th of March is presented in Fig. 5c. Thus, air masses that reached boundary layer height at Quito originated from lower levels of the atmosphere over the Colombian Amazon, where there were abundant fires according to the MODIS image in Fig. 5a. It is important to acknowledge the difficulty of performing backtrajectory modeling to boundary layer levels over very complex terrain such as Quito. Taking this caveat into account, the starting height for the lower backtrajectory was set at 1500 m. a.g.l., this is 500 m above the mountain ridge altitude. Although more modeling and experimental work is necessary to track the origin of polluted air masses, the peculiar circumstances of the COVID-lockdown, that eliminated urban pollution, suggest that these high AOD and PM_2.5_ events over Quito are due to long-range transport.

With respect to the biomass burning season in the Brazilian Amazon in July to November, it has been extensively studied to the point of being generally regarded as the biomass burning season in South America^35,49^. As discussed before, this is not the only region in South America that seasonally undergoes biomass burning. For this reason, in this section we only focus on case studies that deal with the February–March biomass burning season to highlight its importance and emphasize its regional impact over northern South America.

### Aerosol properties and composition

**Figure 6:**
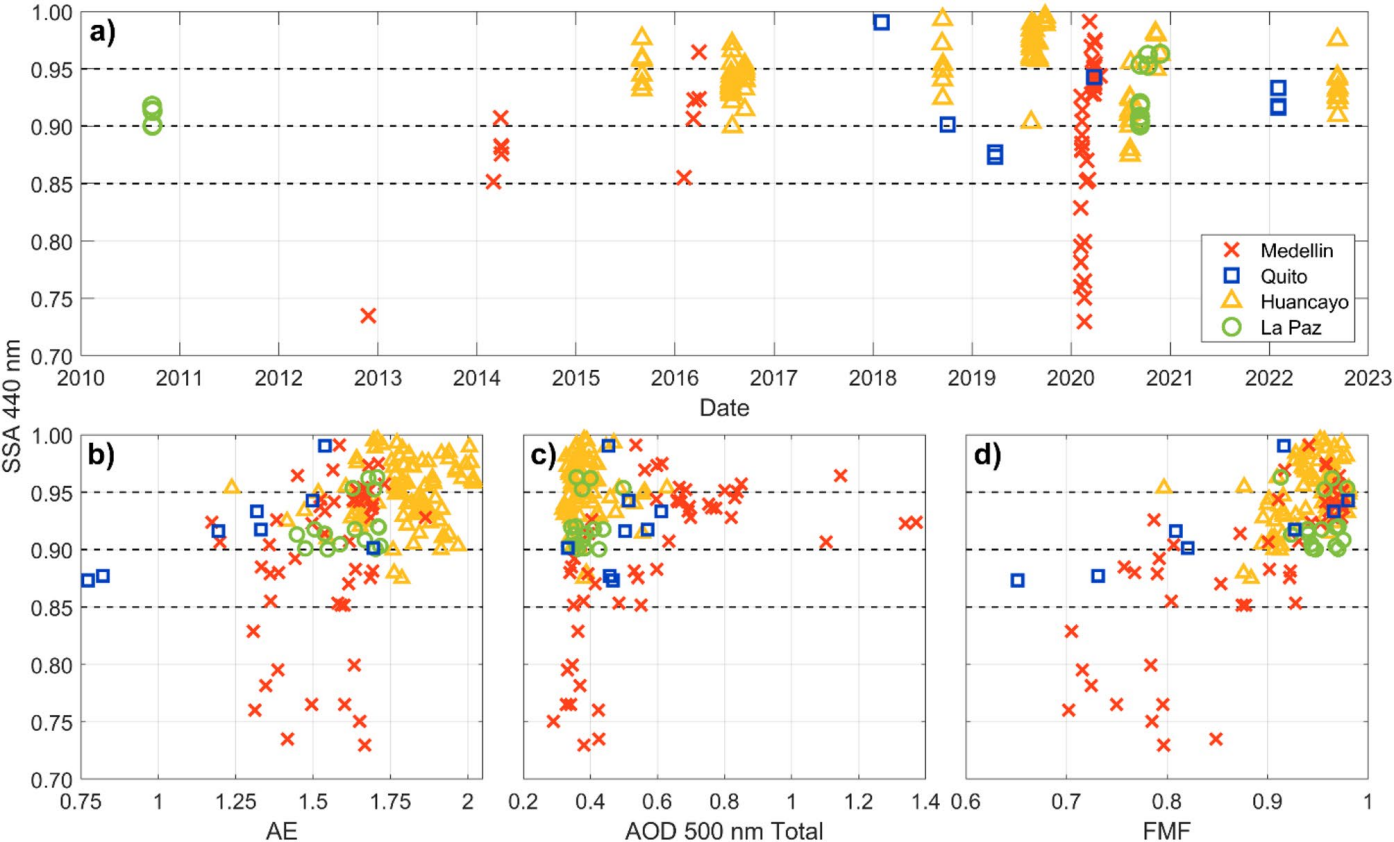
(**a**) Time series of AERONET Version 3 Level 2.0 SSA (single scattering albedo) retrievals available from hybrid and almucantar retrievals at Medellin (red crosses), Quito (blue squares), Huancayo (yellow triangles), and La Paz (green circles). (**b**) SSA at each station (same colors) as a function of Angström Exponent (AE). (**c**) SSA as a function of total AOD 500 nm. (**d**) SSA as a function of FMF (fine mode fraction) at 500 nm.

The aerosol type can be determined considering the simple relationship between aerosol absorption and relative size of the particles with the greatest separation of general particle types using the SSA and AE^50^. With additional information, SSA at 440 nm, FMF at 500 nm and the magnitude of the AE were used to identify aerosol types^43,51^ at each location during times when SSA retrievals were possible, as depicted by the time series in Fig. 6a. As indicated earlier, conditions for almucantar and hybrid retrievals of aerosol properties limit the number of data points available for analysis. Thus, the number of available retrievals (merged hybrid and almucantar) are 52, 8, 92 and 16 for Medellin, Quito, Huancayo, and La Paz, respectively.

At Medellin, most retrievals (51) occurred from February to April 2020 (Fig. 6 and Fig. S6). Only 10 retrievals have characteristics of fine non-absorbing urban aerosols (SSA > 0.96 and FMF > 0.9). The rest corresponds to black carbon aerosols (BC) with FMF > 0.7, whose absorptivity is high (SSA < 0.85, 10 cases), moderate (0.85 < SSA < 0.9, 12 cases), and low (0.9 < SSA < 0.95, 20 cases)^43^. From these aerosol type thresholds, 41 cases at Medellin are identified as black carbon dominated aerosol mixtures. The source may be partly from burning Orinoco region savanna grasses, which can combust rapidly and inefficiently under drier conditions, similar to fires in grasslands of Africa and Australia^52,53^. The biomass burning nature of aerosols in these retrievals is also evident from the high AE (> 1.25) along with SSA 440 nm generally below 0.95^51^ and AOD between 0.3 and 1.4 (Fig. 6b,c,d). Furthermore, aerosols in these retrievals also show absorbing properties at longer wavelengths (675, 870, and 1020 nm in addition to 440 nm) as shown in Fig. S7. At Quito, the number of samples is not sufficient to perform a conclusive assessment. The ranges within which values of SSA 440 nm, AE, and FMF are distributed (Fig. 6), could be an indicative of the combination of properties that urban aerosols possess, but additional data are needed to better identify aerosol types. At Huancayo, SSA measurements were taken between July and November (Fig. S6). 47% of retrievals correspond to fine (FMF > 0.9) non-absorbing (SSA > 0.95) aerosols with high AE values (0.75–2.05). These observations point towards aerosols of a local anthropogenic origin, which is consistent with PM_2.5_ pollution measurements and chemical characterization that identified nitrate, ammonia, and sulfate fractions discussed earlier^23^. The rest of the retrievals (53%) correspond mostly to fine slightly absorbing aerosols also with high AE values (FMF mostly greater than 0.9, SSA: 0.9–0.95, AE: 1.25–2) indicative of transported biomass burning smoke. Mean SSA values at all wavelengths are depicted in Fig. S7. At La Paz, the contribution of absorbing aerosols from transport of biomass burning smoke is evident as SSA 440 nm values are clustered within 0.9–0.95, values of AE are high (1.3–1.75), FMF > 0.9, and most of these data were retrieved in September (Fig. 6). Finally, these were also absorbing aerosols across longer wavelengths (Fig. S7).

To complement the previous retrieval analysis with an extended data set, we present annual cycles of aerosol composition from MERRA-2 monthly means (2006–2022) in Fig. S8. At the moment, we intend to use this MERRA-2 summary as a qualitative indicator of the types of aerosols that are distributed latitudinally over the tropical Andes in different seasons. A more quantitative assessment would require testing the MERRA-2 apportionment of individual aerosol contributions to AOT 550 nm with extended time series of AERONET aerosol optical properties. Aside from these limitations, we find that major features in annual cycles of MERRA-2 aerosol extinction AOT 550 nm by individual aerosols (Fig. S8) relate to findings discussed previously.

In contrast to the aerosol type technique above, MERRA-2 at Medellin indicates organic carbon rather than black carbon dominates in March due to the transport of biomass burning smoke. In general, organic carbon particles contain carbon content with highly scattering properties while black carbon particles are more strongly absorbing particles. The literature indicates that MERRA-2 black carbon and organic carbon emissions datasets may not include some local sources of combustion aerosols^54^. Thus, the MERRA-2 determination of organic particles as the dominant species may be partly in error due to unaccounted emission sources in the Orinoco region.

The local contribution of sulfate aerosols is the second largest at Medellin (20–60%, Fig. S8) with higher loadings during the first and last trimesters, when humidity is higher and can contribute to hygroscopic growth of urban aerosols (Fig. 3b). Sea salt is almost as important as SO_4_ during the first trimester, as the valley where Medellin is situated is on the path of the easterly flow and trade winds that come from the Caribbean sea^55^. Medellin is also affected by intrusion of Sahara dust advected from Africa^56,57^, which explains the dust contribution in Fig. S8. Recent work that analyzed metals in PM_10_ in this region found carbonaceous fractions and metals that were attributed to fuel combustion^58^. Also, sodium and calcium ions were quantified, which are connected to sea salt, although the study focuses on the toxicity aspect of metals rather than in ions from natural sources.

At Quito, the largest contributions are sulfate aerosols and organic carbon with maxima in March and November, which coincides with maxima in the PM_2.5_ and AOD signals (Fig. S8). Currently, we attribute this composition to urban aerosols, although it is possible that a portion of the organic carbon in March comes from long-range transport. These signals are also consistent with the time of the year when the air holds more water vapor, which has an effect in the growth of urban aerosols. A 2020 study on the chemical characterization of PM_10_ in Quito found sulfate, nitrate, and ammonia ions that were directly connected to heterogenous formation of secondary aerosols from urban precursors^59^.

At Huancayo and La Paz, the organic carbon signals peak in September (Fig. S8), which coincides with the peak of biomass burning smoke transport, although at Huancayo the signal is twice as large. There is a background signal of sulfate aerosols that is higher at Huancayo and slightly increases in September. The presence of sulfate aerosols and other urban fractions in PM_2.5_ at Huancayo was discussed earlier^23^. In Bolivia, a recent study characterized the chemical composition of aerosols at the Chacaltaya station^60^. These measurements could serve as reference, although the Chacaltaya station is about 20 km north of La Paz and at higher altitude (5240 m a.s.l.). The authors of the study found urban traces from La Paz traffic in the form of elemental carbon and nitrate ions combined with the tracer oxalate. At the high-altitude locations of Quito, Huancayo, and La Paz, the sea salt contribution is less important for being secluded within the Andes. Aside from coarse particles of local origin, these locations are not affected by large advection of Saharan dust as opposed to the northern Medellin location.

### Precipitable water vapor distribution

**Figure 7:**
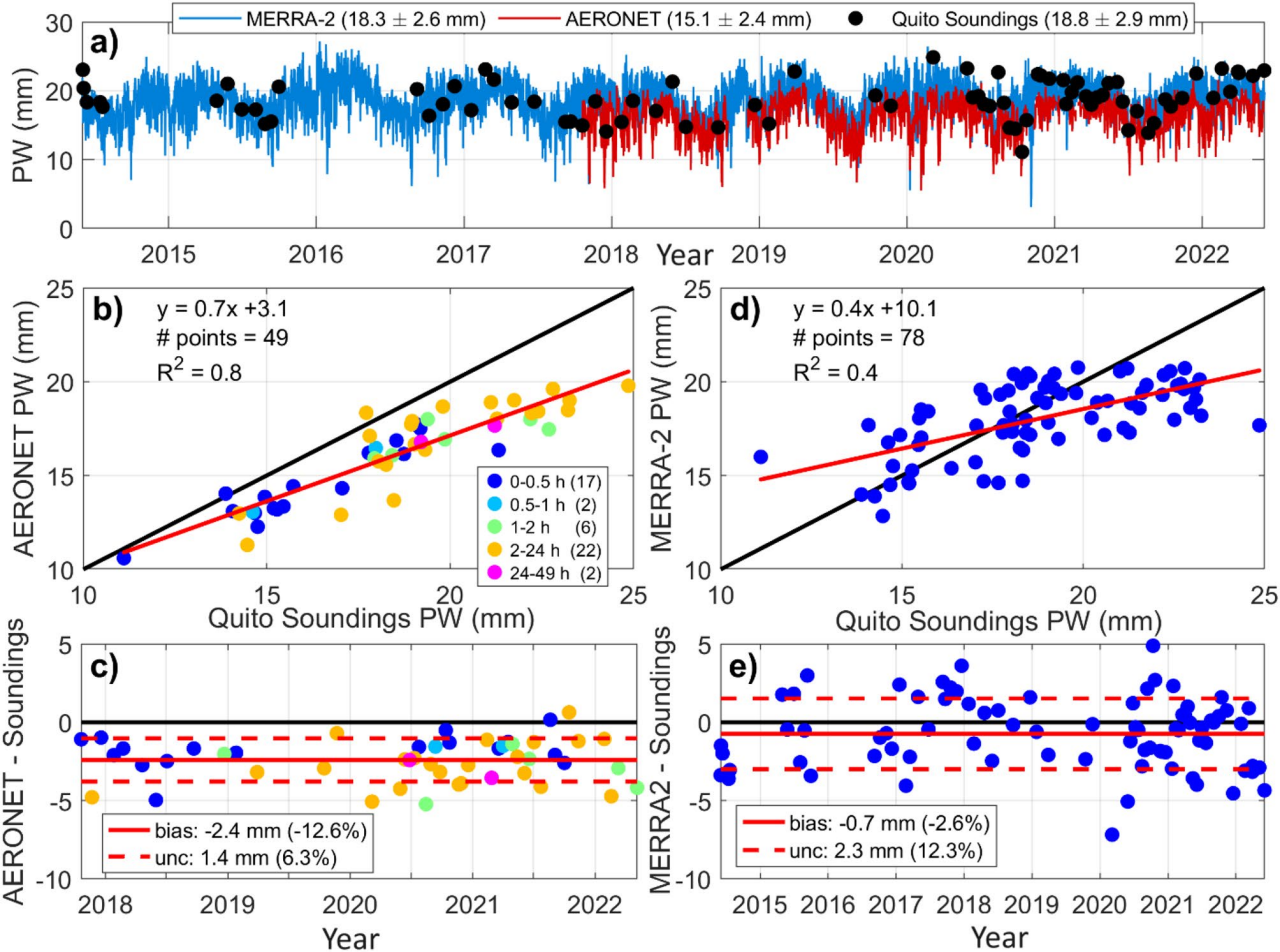
(**a**) Time series of columnar water vapor from soundings at Quito (black dots) overlapped with columnar PW (precipitable water vapor) from AERONET Version 3 Level 2.0 (5-min data, red line), and MERRA-2 Total PW (1-h data, blue line). (**b**) 1:1 comparison and linear regression between AERONET PW (interpolated to sounding time stamp) and sounding measurements. The time window for comparisons is indicated by colored dots with the number of data points in parentheses. (**c**) Time series of differentials between AERONET and sounding columnar water vapor with quantification of mean bias (mean of differentials) and uncertainty (1 standard deviation). (**d**) and (**e**) are same as (**b**) and (**c**), but for comparisons between 1-h MERRA-2 Total PW (interpolated to sounding time stamp) and sounding measurements.

Columnar measurements of precipitable water vapor are snapshots of the amount of moisture that the atmosphere holds at a given time expressed as the depth of precipitation (mm) that would result if all the water vapor in the atmospheric column would condense. Not all water vapor condenses as precipitation, but the effective amount of rainfall directly depends on the atmospheric water vapor column and impacts the water cycle within a region. AERONET columnar measurements provide means to characterize PW in the tropical Andes. At the same time, balloon-borne measurements of the water vapor column from Quito provide a check for AERONET measurements. Comparisons between AERONET columnar measurements of PW and the water vapor column from sounding data (integrating water vapor mixing ratio from the surface up to 17 km a.s.l.) are presented as time series in Fig. 7a. Out of 78 in situ measurements, 49 overlap with AERONET. Hence, this analysis was complemented with MERRA-2 total precipitable water vapor (1-h) that overlaps with the entirety of sounding and AERONET measurements. The main temporal features observable in the time series such as times when PW increases or decreases are consistent across data sets, but there are differences that are quantified below.

A linear fit between AERONET PW and sounding data is presented in Fig. 7b (R^2^ = 0.8). Differences between AERONET and soundings are presented in Fig. 7c. Statistics on differentials indicate that the mean bias of AERONET columnar measurements with respect to soundings is − 12.6%, while the 1–σ uncertainty is 6.3%. Thus, AERONET underpredicts in situ measurements, which is also reflected in the mean PW of the time series (15.1 ± 2.4 mm vs.18.8 ± 2.9 mm, Fig. 7a). Previous research done at lower elevation demonstrated that AERONET compares within stated uncertainty of 10% (Methods) using radiosonde and microwave radiometer water vapor data when excluding dry conditions^61,62^. For example, under the dry wintertime in the Northern Hemisphere, the Cimel sun photometer measurements could be lower than soundings by 25%^61^. The comparisons presented in this work were done at a high elevation station where the atmospheric water vapor column is rather dry when compared to other sites where AERONET PW has been evaluated^63^. Hence, it is possible that the AERONET PW dry bias has an elevation dependency that needs to be further explored in the future. Analyzing a larger number of coincident AERONET and sounding time series will help refine the relationship of AERONET PW to radiosonde PW for the Quito region. A more complete evaluation will require additional sounding data from other stations over the Andes.

With respect to MERRA-2 Total precipitable water vapor is less correlated with respect to sounding columnar measurements than AERONET, as indicated in Fig. 7d (R^2^ = 0.4). Furthermore, uncertainty is higher (12%), but bias is lower (-2.6%) as shown in Fig. 7e. As a result of a low overall bias, the mean of the MERRA-2 PW (18.3 ± 2.6 mm) is close in magnitude to the mean PW measured from soundings at the Quito station (18.8 ± 2.9 mm). This match is due to statistical smoothing of individual differences, but the linear regression shows poor correlation for point comparisons. As in the case of AERONET, additional work is needed to refine the relationship of MERRA-2 PW to radiosonde PW. The consistency of MERRA-2 data over time and the fact the bulk of the data as a mean of observations yields a result close to the mean of in situ observations is advantageous despite the caveats, as it allows expanding analysis to other Andes regions where observations are scarce.

**Figure 8:**
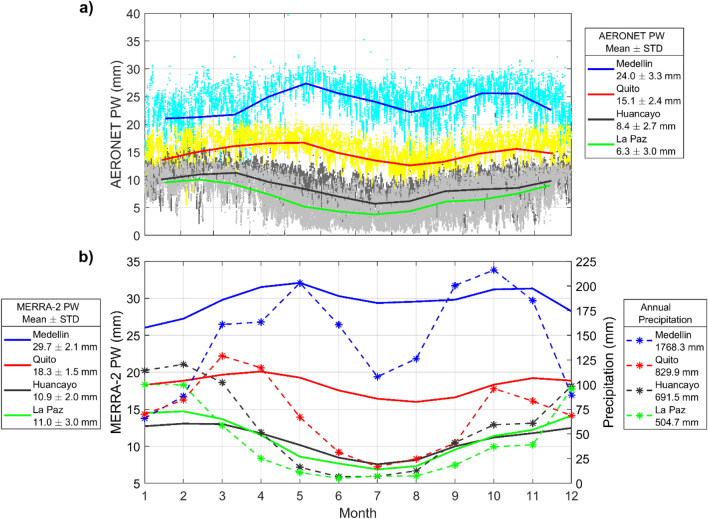
(**a**) AERONET Version 3 Level 2.0 precipitable water vapor column at Medellin (blue), Quito (red), Huancayo (black), and La Paz (green). Colored dots are 1-h data, while solid lines are annual cycles from monthly means. Label indicates mean PW + /– 1 standard deviation (STD). (**b**) Solid lines are annual cycles from MERRA-2 Total precipitable water vapor monthly means from 2006 to 2022 and with same colors for each station as in (**a**). Dashed lines and stars are monthly means of cumulative precipitation from surface measurements. Periods of data availability for each case are indicated in the Methods.

The effect of station altitude in PW is evident when AERONET observations are overlapped as annual cycles (Fig. 8a). Huancayo and La Paz, sited respectively at 3313 and 3439 m a.s.l., have the driest water vapor columns (8.4 ± 2.7 mm and 6.3 ± 3 mm, respectively), while Quito (2800 m a.s.l.) and Medellín (1495 m a.s.l.) have increasing PW (15.1 ± 2.4 mm and 24 ± 3.3 mm). Average AERONET PW measurements could be 15% lower than soundings and MERRA-2 data if we take as reference the Quito comparison. Future work needs to incorporate independent in situ measurement of the water vapor column at each station to better evaluate both AERONET and MERRA-2 PW data. However, from an observational perspective, this characterization of columnar water vapor content can help interpret precipitation measurements at the surface, although a thorough characterization of precipitation patterns needs to consider a synoptic analysis that is beyond the scope of this work and can be found elsewhere^51^.

Given that there are data gaps in the AERONET PW time series and sampling periods do not entirely overlap, we used MERRA-2 monthly means of PW from 2006 to 2022 along with precipitation measurements at each station for the same period, when available, to characterize and relate both quantities. Thus, Fig. 8b shows annual cycles from both data sources. The MERRA-2 PW means in Fig. 8b are not directly comparable with AERONET means in Fig. 8a because the length of the time series and the temporal resolution are different, in addition to the experimental bias discussed earlier. However, we take advantage of the availability and consistency of MERRA-2 data and its overall good performance at one of the stations to interpret extended precipitation time series.

From Fig. 8b, the Medellin and Quito regions have resembling PW patterns and two marked rainy seasons with maxima in May and October (Medellin) and March–April and October–November (Quito). On the other hand, the pattern of PW annual cycles at Huancayo and La Paz are similar as well as the rainy season that starts in November and extends until February. The magnitudes of PW and precipitation are greater from north to south. Thus, in a year the mean columnar water vapor (MERRA-2) and annual rainfall at stations from north to south are: 29.7 mm and 1768 mm at Medellin, 18.3 mm and 830 mm at Quito, 10.9 mm and 692 mm at Huancayo, and 11 mm and 505 mm at La Paz. At mountainous regions usually there is spatial variability in precipitation due orographic uplifting. For example, at the north of Quito annual rainfall could be about 1000 mm. For the characterization presented in this work, only data at AERONET locations or close were evaluated. Additional assessments need to consider spatial variability in precipitation.

The collective analysis of mean PW and rainfall presented in this work depicts the latitudinal and temporal distribution of these quantities over the Andean tropics. Future work is needed to look at trends of both quantities in connection with climate predictions that project increasing extreme events of precipitation in northeastern South America and increasing droughts at southern latitudes in decades to come.

### Regional summary

**Figure 9:**
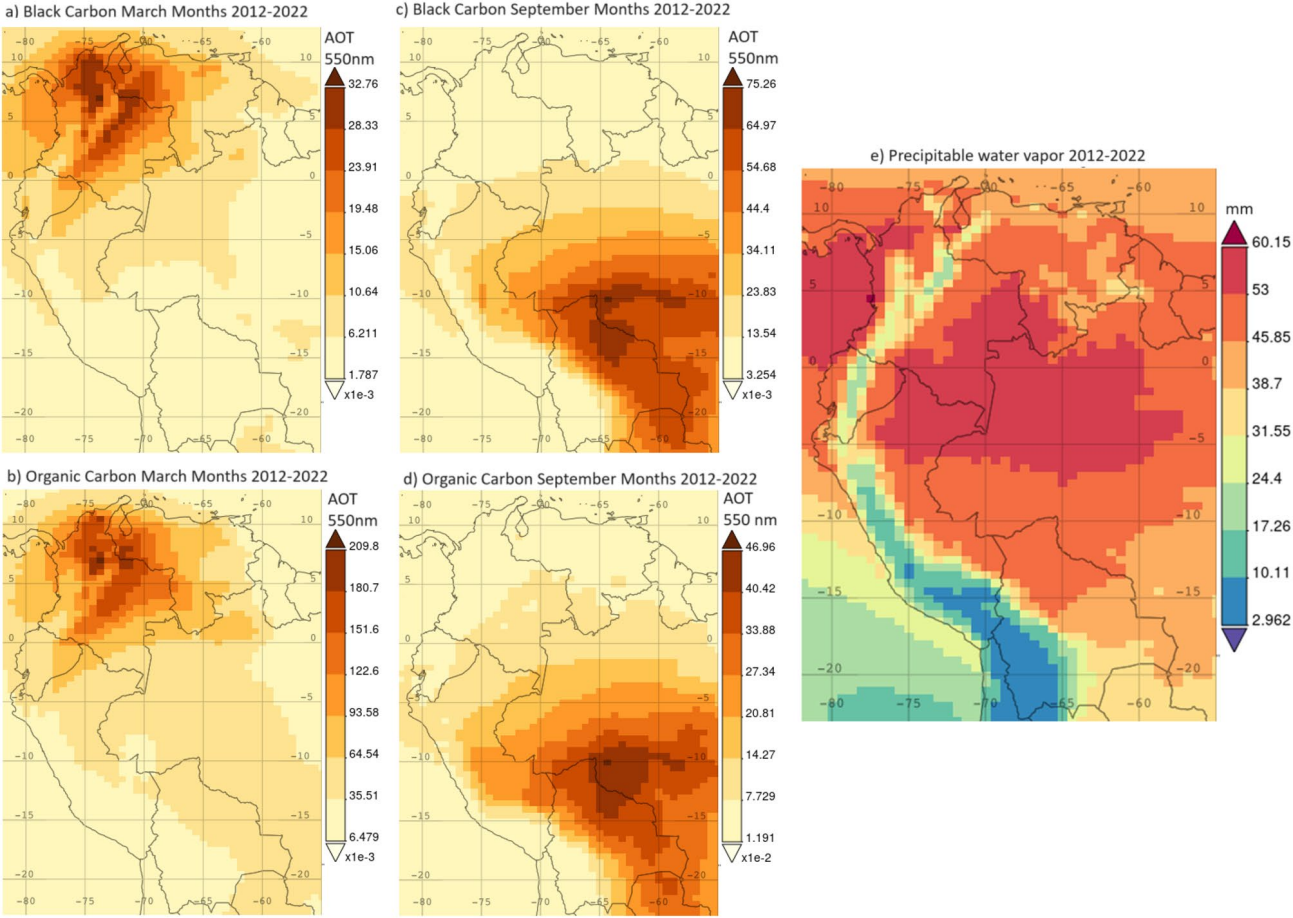
Regional maps of MERRA-2 (AOT 550 nm) as recurring averages between 2012 and 2022 of: (**a**) Black carbon, March months (**b**) Organic carbon, March months, (**c**) Black carbon, September months, (**d**) Organic carbon, September months. (**e**) Time-average regional map of MERRA-2 PW between 2012 and 2022. Maps were generated online from the Giovanni NASA website (https://giovanni.gsfc.nasa.gov/giovanni/).

The regional and temporal distribution of aerosols over the tropical Andean region are significantly influenced by biomass burning activities and topography. A bimodal distribution of biomass burning seasons exists within a year in South America systematically impacting the Andean tropics. Further, the Andes topography influences aerosol distribution. To illustrate these observations, MERRA-2 black and organic carbon (extinction AOT 550 nm) during months when peak AERONET fine-mode AOD signals are observed at Andean sites are mapped in Fig. 9a to d as recurring averages over the past ten years (2012–2022) for the months of March and September. Thus, Andean locations at Colombian valleys and highlands are directly affected by the March biomass burning season. The altitude and intricate topography of the Andes mountains block massive smoke transport into the Ecuadorian highlands, although it is possible that a fraction of the March AOD signal detected by the Quito AERONET instrument is due to cross-Andean transport from Colombian fires. Highland locations in Peru and Bolivia are not impacted by the March season. In contrast, the peak impact observed over central Peru and the Bolivian highlands occurs in September, when the AERONET fine-mode AOD increases due to the massive biomass burning season in the Amazon, although most of the smoke transport is curved by the Andes elevation.

The MERRA-2 aerosol composition partitioning for the multi-year annual average is shown in Fig. S9. It is important to remark that the annual mean of the regional distribution of black and organic carbon aerosols (Fig. S9a and b) incorporates the effects of two biomass burning seasons without distinguishing between the times of the year when they occur. Hence, discussing biomass burning impacts in South America using only multi-year annual averages of aerosol-related quantities could be misleading because the magnitude of the biomass burning season in the Brazilian Amazon overshadows the biomass burning season over northern South America. Figure S9c to e shows the regional distribution of sulfate aerosols, dust, and sea salt. Sulfates are distributed along the Pacific Ocean coastline of northwest South America with higher magnitudes near urban centers in Colombia, Ecuador, and Peru. Dust is mostly distributed over northeast South America due to the intercontinental transport from the Saharan region. Likewise, sea salt is mostly present over northern Colombia as it arrives with the northeasterly flow from the Caribbean Ocean.

In regard to precipitable water vapor, a 10-year annual average of MERRA-2 PW over the region is shown as a map in Fig. 9e. The silhouette of the Andes mountain chain is evident from PW data depicted in the region across 30° of latitude from north to south. A major reason for this distribution is the topographic factor that induces higher PW at lower elevation regions at northern Andean locations and progressively lower PW over the highlands in Ecuador and central Peru, while Andean areas in southern Peru and Bolivia are the driest. This regional distribution along with levels of PW and annual rainfall quantified in the previous section come together as a diagnosis of the current status of PW in the Andean tropics whose changes need to be closely tracked in time. Particularly, tropospheric water vapor is distributed from the Amazon to the central Andes and south of the equator^55^, but alarming news of drought in the Amazon^64^ could seriously affect rainfall amounts in Andean countries. On the other hand, northwestern regions are projected to experience more frequent episodes of extreme precipitation^4^, which combined with the very complex topography increase the risk of floods and landslides that impact human lives and livelihoods^1^.

## Conclusions

The tropical Andes is a high-altitude inhabited region that provides a unique divide between the South American Pacific coast and the Amazon basin, and whose topography directly influences the regional distribution of aerosols and water vapor.

In regard to aerosol composition, the dominant mixture of urban aerosols and biomass burning smoke aerosols become distributed with different intensities within a year from north to south between latitudes 6° N and 16.5° S. The orientation and altitude of the Andes cordillera play a role at funneling or blocking transport of biomass burning smoke into Andean valleys or highlands during the two fire seasons that systematically impact South America. The peak of the first season occurs in February–March, north of the Colombian Andes, at the Colombian-Venezuelan border, and the grasslands by the Orinoco River basin. This first season is less known or studied than the biomass burning season in the Brazilian Amazon, which runs from July to October and peaks in September. The footprint of these influences is evident in AERONET AOD annual cycles that share partial similarities between stations Medellin (Colombia) and Quito (Ecuador) during the first trimester, although the Medellin signal is substantially higher. Meanwhile, AOD annual cycles at stations Huancayo (Peru) and La Paz (Bolivia) share similarities during the third and fourth trimesters.

The Andes cordillera in northern Colombia lays parallel to the burning region and is on the path of the easterly flow that transports biomass burning smoke into populated Andean valleys deteriorating their air quality during the first moths of the year. AERONET total AOD 500 nm (5-min) at the Medellin station in March can reach values close to 2.0 and local measurements (1-h) of PM_2.5_ rise above 60 µg m^–3^. As a result of this major influence, the average fine-mode AOD and surface PM_2.5_ peak in March at 0.5 and 56 µg m^–3^. Both quantities correlate linearly in a year for simultaneous measurements. Further, in Medellin, these aerosol particles were determined by AERONET SSA to be highly absorbing such as from black carbon particles rather than organic particles identified by MERRA-2. This difference may be due partly to unaccounted biomass burning emissions by MERRA-2 in the Orinoco region.

At the equatorial Quito station, the urban footprint dominates in the AERONET AOD annual cycle and linearly correlates with the annual PM_2.5_ cycle for simultaneous measurements. Both quantities peak in March and November. In these months the fine-mode AOD 500 nm and PM_2.5_ are about 0.14 and 15 µg m^–3^, on average. Towns in the Ecuadorian highlands are more secluded within the Andes mountains in a way that a blocking effect due to the Andes altitude is more effective at restraining the direct impact of biomass burning smoke transport, especially in the first trimester. However, it is possible that events of long-range transport from burning areas in eastern Colombia in March reach Andean locations in the north of Ecuador. This cross-Andean transport was observed during a high AOD and PM_2.5_ event at Quito in the absence of urban pollution during the COVID-19 lockdown. As biomass burning seasons continue to intensify, the effect of long-range transport needs to be monitored over these regions that currently are not impacted systematically by these events. In the future, chemical analyses to track carbonaceous fractions of PM_2.5_ to biomass burning smoke aerosols would help interpret AERONET aerosol optical properties and aerosol apportionment in products such as MERRA-2 AOT 550 nm.

At southern locations, the Peruvian and Bolivian Andes curb the bulk of the smoke transport from burning areas in the Amazon. However, background fine-mode AOD 500 nm does increase at AERONET stations Huancayo (from 0.05 to 0.12) and La Paz (from 0.05 to 0.1) in September. From SSA and AE measurements, a combination of aerosols of local sources in addition to biomass burning smoke make the atmospheric column at Huancayo more polluted with non-absorbing aerosols than at La Paz.

With respect to PW, AERONET columnar measurements agree with sounding data within expected levels of uncertainty. The atmospheric column generally holds more moisture from north to south as a direct effect of altitude, although synoptic circulation and moisture advection were not analyzed in this work. Thus, at Medellin (6.26° N, 1500 m. a.s.l.) the mean PW is almost 30 mm and annual precipitation is 1768 mm. At the Quito station, one thousand meters higher and on the equator, the mean PW is 18.3 mm and annual precipitation is about 900 mm. At Huancayo and La Paz (12° and 16.5° S at 3300 and higher than 3400 m a.s.l.), PW is about 11 mm, but annual precipitation rates are about 700 and 500 mm, respectively. This characterization of the current distribution and magnitude of the water vapor column in connection to annual precipitation contributes as an underlying basis for future studies that analyze the evolution of hydrological climate risks such as extreme precipitation and droughts in the region.

### Supplementary Information

Supplementary Information.

## Data Availability

Below sources where data are available: AERONET data: https://aeronet.gsfc.nasa.gov/. Quito soundings: https://observaciones-iia.usfq.edu.ec/. under ‘USFQ Data\Sondeos\EMA_Soundings’. Quito PM_2.5_ and humidity: Table S2. Medellín PM_2.5_ and meteorological data: https://siata.gov.co/siata_nuevo. Huancayo precipitation data: climatology in Table S3. Bolivia precipitation data: https://www.ine.gob.bo/index.php/medio-ambiente/clima-y-atmosfera/. MERRA-2 PW and AOT data: https://giovanni.gsfc.nasa.gov/giovanni/. HYSPLIT archive trajectories: https://www.ready.noaa.gov/HYSPLIT.php. Satellite images from MODIS and VIIRS: https://worldview.earthdata.nasa.gov.
