# Supplementary material for: Latitudinal and temporal distribution of aerosols and precipitable water vapor in the tropical Andes from AERONET, sounding, and MERRA-2 data
Source: Sci Rep. 2024 Jan 9;14:897. doi: 10.1038/s41598-024-51247-9 (PMC10776852; doi:10.1038/s41598-024-51247-9)
Supplement: Supplementary file 1 — Supplementary Information. [file 41598_2024_51247_MOESM1_ESM.docx]

**Supplementary Information**

**Latitudinal and temporal distribution of aerosols and precipitable water vapor in the tropical Andes from AERONET, sounding, and MERRA-2 data**

María Cazorla^1*^, David M. Giles^2,3^, Edgar Herrera^1^, Luis Suárez^4^, Rene Estevan^4^, Marcos Andrade^5^, Álvaro Bastidas^6^

^1^Universidad San Francisco de Quito USFQ, Instituto de Investigaciones Atmosféricas, Quito, Ecuador, ^2^Science Systems and Applications, Inc. (SSAI), ^3^NASA Goddard Space Flight Center (GSFC), ^4^Instituto Geofísico del Perú. ^5^Universidad Mayor de San Andrés, Bolivia. ^6^Universidad Nacional de Colombia – Medellín.

*mcazorla@usfq.edu.ec

**Supplementary Tables**

**Table S1** Total number of data points from AERONET products per variable and station.

| **Variable** | **Total number of points** | | | | **AERONET Product** |
| --- | --- | --- | --- | --- | --- |
|  | **Medellín** | **Quito** | **Huancayo** | **La Paz** |  |
| PW | 21107 | 46086 | 116463 | 97562 | Direct Sun Measurements (DSM) |
| AOD, FMF | 21004 | 45902 | 116380 | 97326 | Spectral Deconvolution Algorithm from DSM |
| Size Distribution, AE | 114 | 544 | 2734 | 2653 | Aerosol Inversion from Sky Radiance Measurements (Almucantar) |
|  | 62 | 516 | 3020 | 1159 | Aerosol Inversion from Sky Radiance Measurements (Hybrid) |
|  | 176 | 1060 | 5754 | 3812 | Total (Almucantar + Hybrid) |
| SSA | 30 | 7 | 54 | 8 | Aerosol Inversion from Sky Radiance Measurements (Almucantar) |
|  | 22 | 1 | 38 | 8 | Aerosol Inversion from Sky Radiance Measurements (Hybrid) |
|  | 52 | 8 | 92 | 16 | Total (Almucantar + Hybrid) |

**Table S2** One-month averages of PM_2.5_ and water vapor mixing ratio (q) calculated from measurements at a station located 4 km away from EMA USFQ station and run by the Quito’s Secretariat for the Environment (more in Methods). Only PM_2.5_ data that coincided with AERONET measurements at EMA station (daytime) were used to produce averages.

| **Date** | **PM_2.5_** | **q** |  | **Date** | **PM_2.5_** | **q** |
| --- | --- | --- | --- | --- | --- | --- |
| **yyyy-mm** | **µgm^-3^** | **g kg^-1^** |  | **yyyy-mm** | **µgm^-3^** | **g kg^-1^** |
| 2017-10 | 15.55 | 9.80 |  | 2020-03 | 13.27 | 10.80 |
| 2017-11 | 18.49 | 9.52 |  | 2020-04 | 10.35 | 10.78 |
| 2017-12 | 16.95 | 9.41 |  | 2020-05 | 9.42 | 10.54 |
| 2018-01 | 13.86 | 9.48 |  | 2020-06 | 10.02 | 9.72 |
| 2018-02 | 13.93 | 10.75 |  | 2020-07 | 9.85 | 8.99 |
| 2018-03 | 15.77 | 10.00 |  | 2020-08 | 10.78 | 8.33 |
| 2018-04 | 12.20 | 10.13 |  | 2020-09 | 12.64 | 8.78 |
| 2018-05 | 13.81 | 10.91 |  | 2020-10 | 11.81 | 8.35 |
| 2018-06 | 13.60 | 9.26 |  | 2020-11 | 18.73 | 9.95 |
| 2018-07 | 11.31 | 8.38 |  | 2020-12 | 13.41 | 10.61 |
| 2018-08 | 13.63 | 7.90 |  | 2021-01 | 9.51 | 10.63 |
| 2018-09 | 16.32 | 8.14 |  | 2021-02 | 13.37 | 10.80 |
| 2018-10 | 28.39 | 8.10 |  | 2021-03 | 12.04 | 10.66 |
| 2018-12 | 12.24 | 9.29 |  | 2021-04 | 11.18 | 10.59 |
| 2019-01 | 10.60 | 9.31 |  | 2021-05 | 10.80 | 10.59 |
| 2019-02 | 14.63 | 11.03 |  | 2021-06 | 12.29 | 10.24 |
| 2019-03 | 15.90 | 10.90 |  | 2021-07 | 9.20 | 8.34 |
| 2019-04 | 16.12 | 11.10 |  | 2021-08 | 12.38 | 8.49 |
| 2019-05 | 14.59 | 10.50 |  | 2021-09 | 9.21 | 8.56 |
| 2019-06 | 13.28 | 9.08 |  | 2021-10 | 13.54 | 10.38 |
| 2019-07 | 12.26 | 8.13 |  | 2021-11 | 11.24 | 10.51 |
| 2019-08 | 13.18 | 7.28 |  | 2021-12 | 13.42 | 11.17 |
| 2019-09 | 14.64 | 7.76 |  | 2022-01 | 15.58 | 10.23 |
| 2019-10 | 13.38 | 9.70 |  | 2022-02 | 19.38 | 10.83 |
| 2019-11 | 10.65 | 10.63 |  | 2022-03 | 13.44 | 10.85 |
| 2019-12 | 13.30 | 10.55 |  | 2022-04 | 13.20 | 10.79 |
| 2020-01 | 10.04 | 9.49 |  | 2022-05 | 13.33 | 10.48 |
| 2020-02 | 14.99 | 9.42 |  |  |  |  |

**Table S3** Precipitation climatology for the periods 1981-2010 and 1991-2020 from measurements at Huayao Station (12.040°S, 75.320°W, 3321 masl). Climatology prepared by the Peruvian Geophysical Institute (IGP, Spanish Acronym) (Giráldez et al., 2020).

|  | **Precipitation (mm)** | |
| --- | --- | --- |
| **Month** | **1981-2010** | **1991-2020** |
| **1** | 118.3 | 114.4 |
| **2** | 128.5 | 120.6 |
| **3** | 104.9 | 102.2 |
| **4** | 53.4 | 51.6 |
| **5** | 14.6 | 16.3 |
| **6** | 8.7 | 6.6 |
| **7** | 7.7 | 7.1 |
| **8** | 15.2 | 12.7 |
| **9** | 40.3 | 41.3 |
| **10** | 68.9 | 59.3 |
| **11** | 66.4 | 60.9 |
| **12** | 94.5 | 98.5 |

**Supplementary Figures**


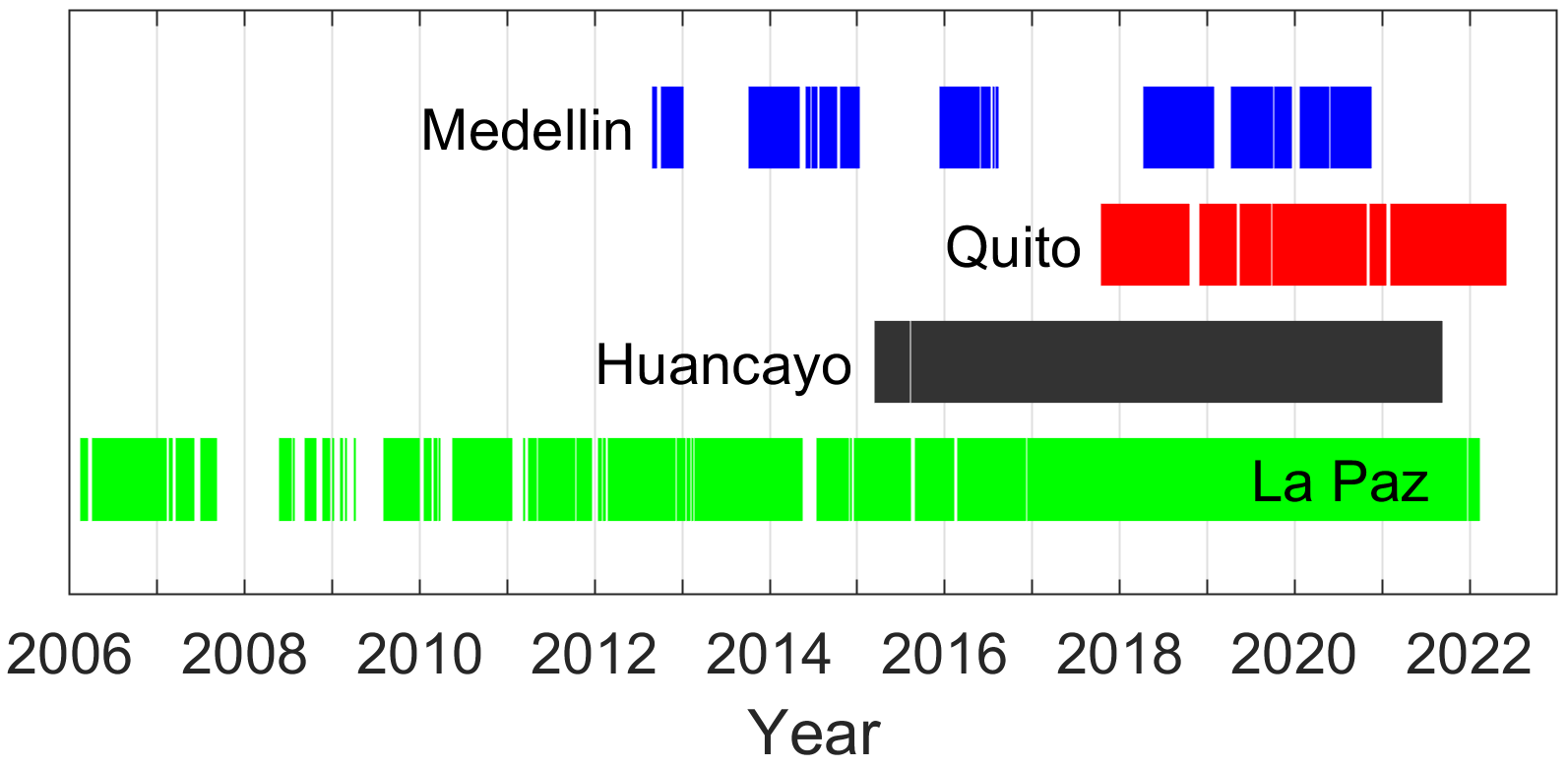


**Figure S1** Timeline of available AERONET Spectral Deconvolution Algorithm (SDA) data at stations Medellín (blue), Quito (red), Huancayo (black) and La Paz (green).


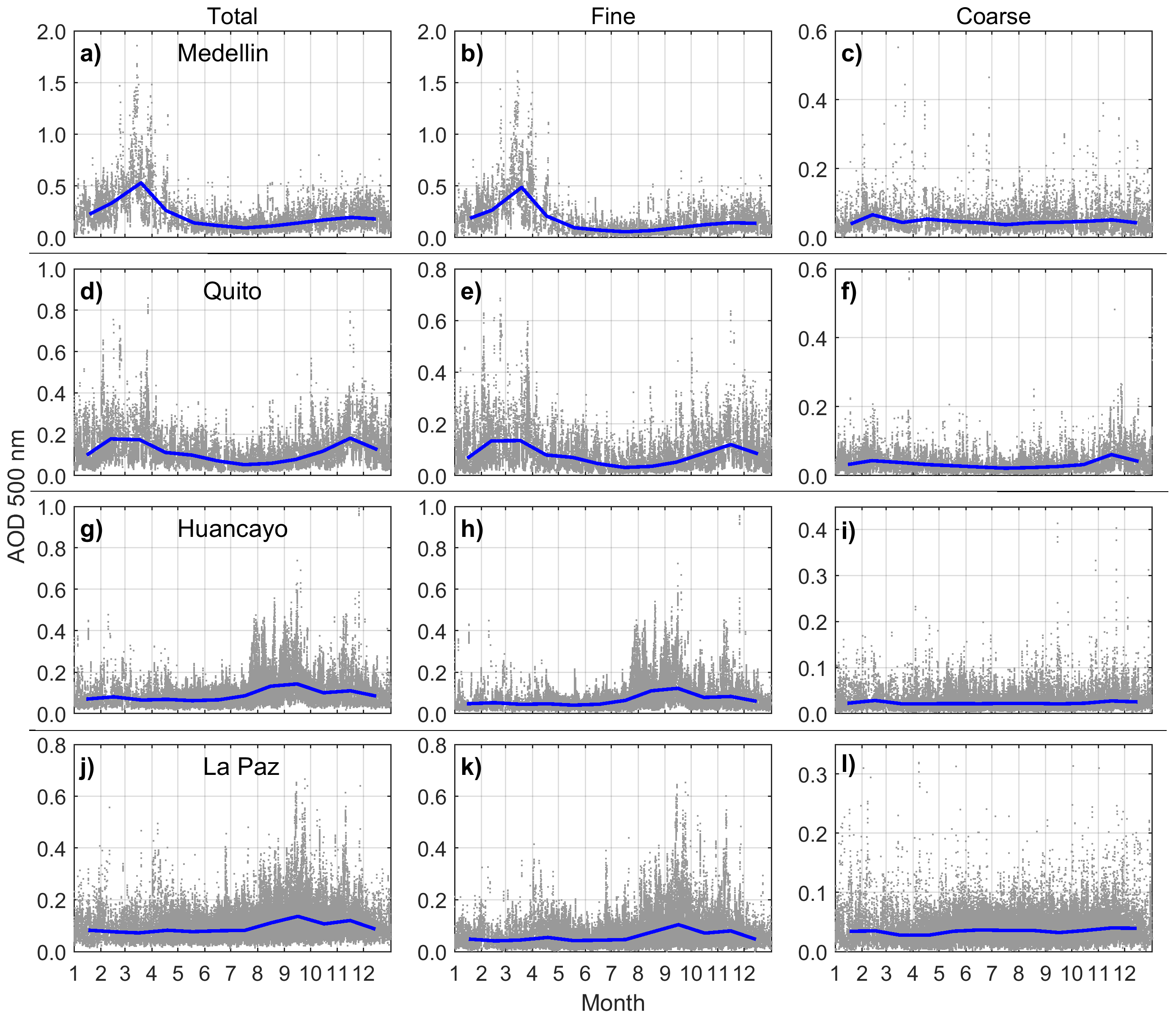


**Figure S2** The 3-minute data (gray points) of AERONET SDA total, fine-mode, and coarse-mode AOD at 500 nm collected at Medellin, Quito, Huancayo, and La Paz, where all four sites overlapped in one year. Solid blue lines depict annual cycles at each station from monthly means.


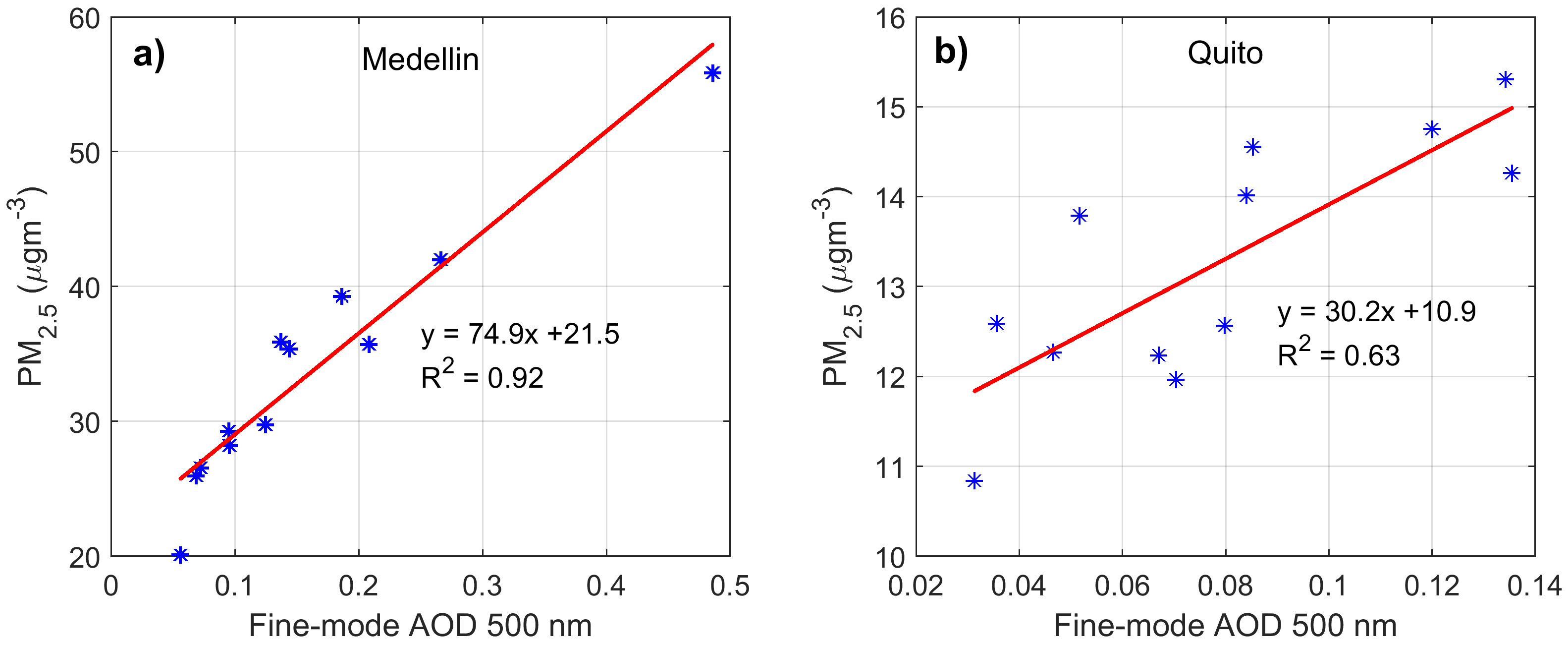


**Figure S3** Linear regression between PM_2.5_ and fine-mode AOD annual cycles at a) Medellin, b) Quito. Only data that were measured at coincident time periods were used to generate annual cycles. These regressions show correlation, but they are not intended to estimate surface PM_2.5_.


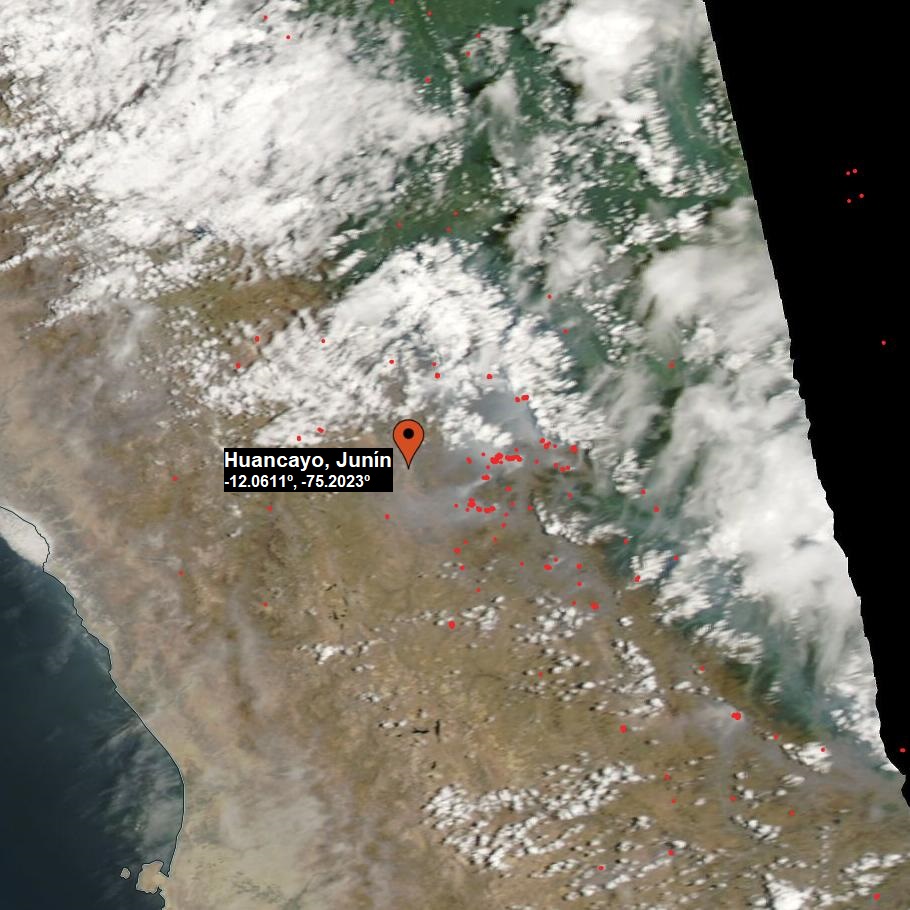


**Figure S4** MODIS (Terra and Aqua) and VIIRS true color image over the Huancayo region with thermal anomalies (in red) indicating fire locations on 24 November 2020.

The image in Figure S4 can be accessed at: <https://worldview.earthdata.nasa.gov/?v=-79.32547556401843,-14.426635720644683,-69.98589266098173,-9.902775251986288&l=Reference_Labels_15m(hidden),Reference_Features_15m(hidden),Coastlines_15m,VIIRS_SNPP_Thermal_Anomalies_375m_All,VIIRS_NOAA20_CorrectedReflectance_TrueColor(hidden),VIIRS_SNPP_CorrectedReflectance_TrueColor(hidden),MODIS_Aqua_CorrectedReflectance_TrueColor,MODIS_Terra_CorrectedReflectance_TrueColor(hidden)&lg=true&s=-75.2023,-12.0611&t=2020-11-24-T13%3A12%3A43Z>


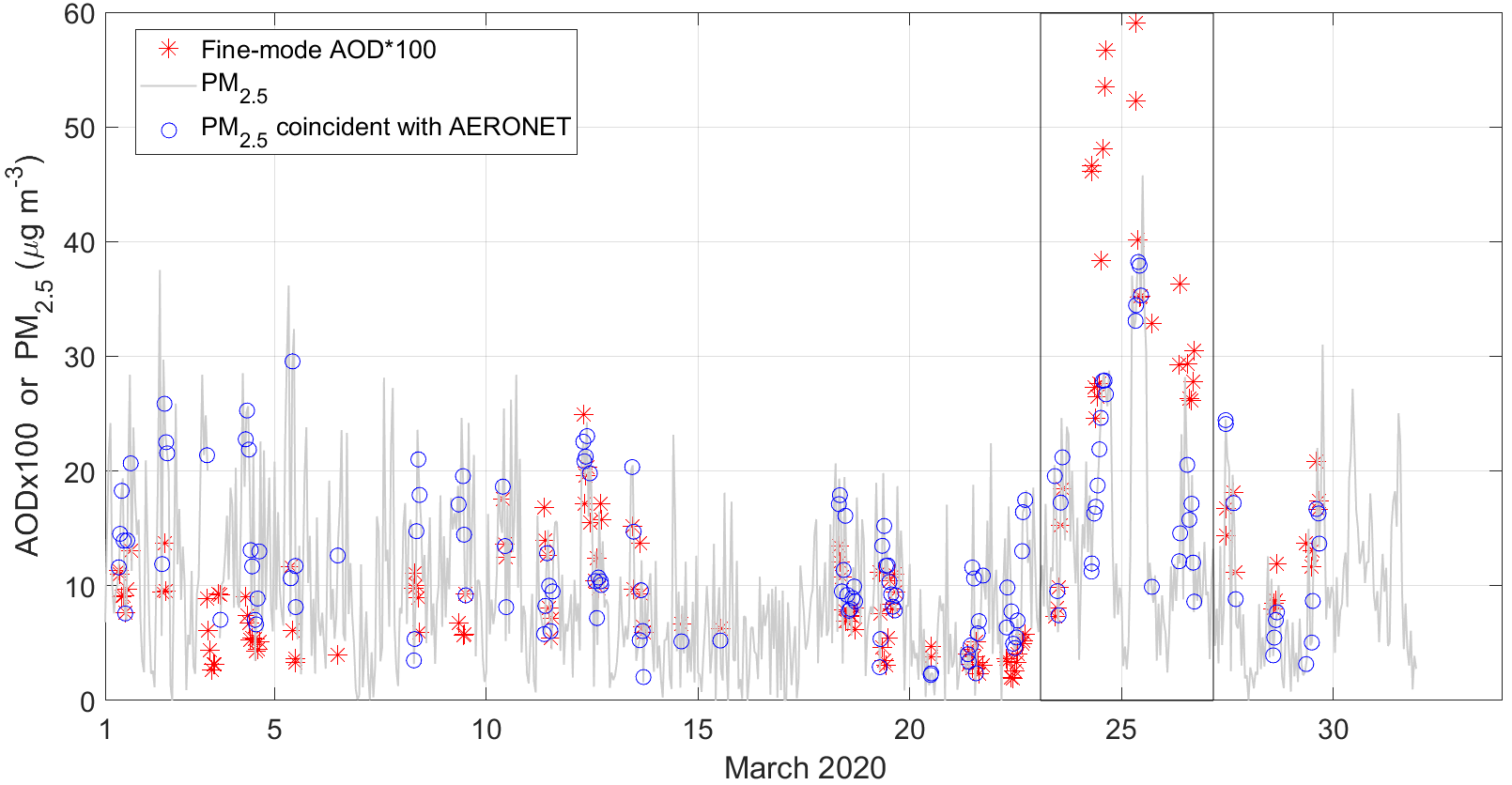


**Figure S5** March 2020 time series of AERONET SDA fine-mode AOD (1-hour averages, red stars) along with continuous PM_2.5_ 1-hour data (gray line) and measurements coincident with AERONET (blue circles).


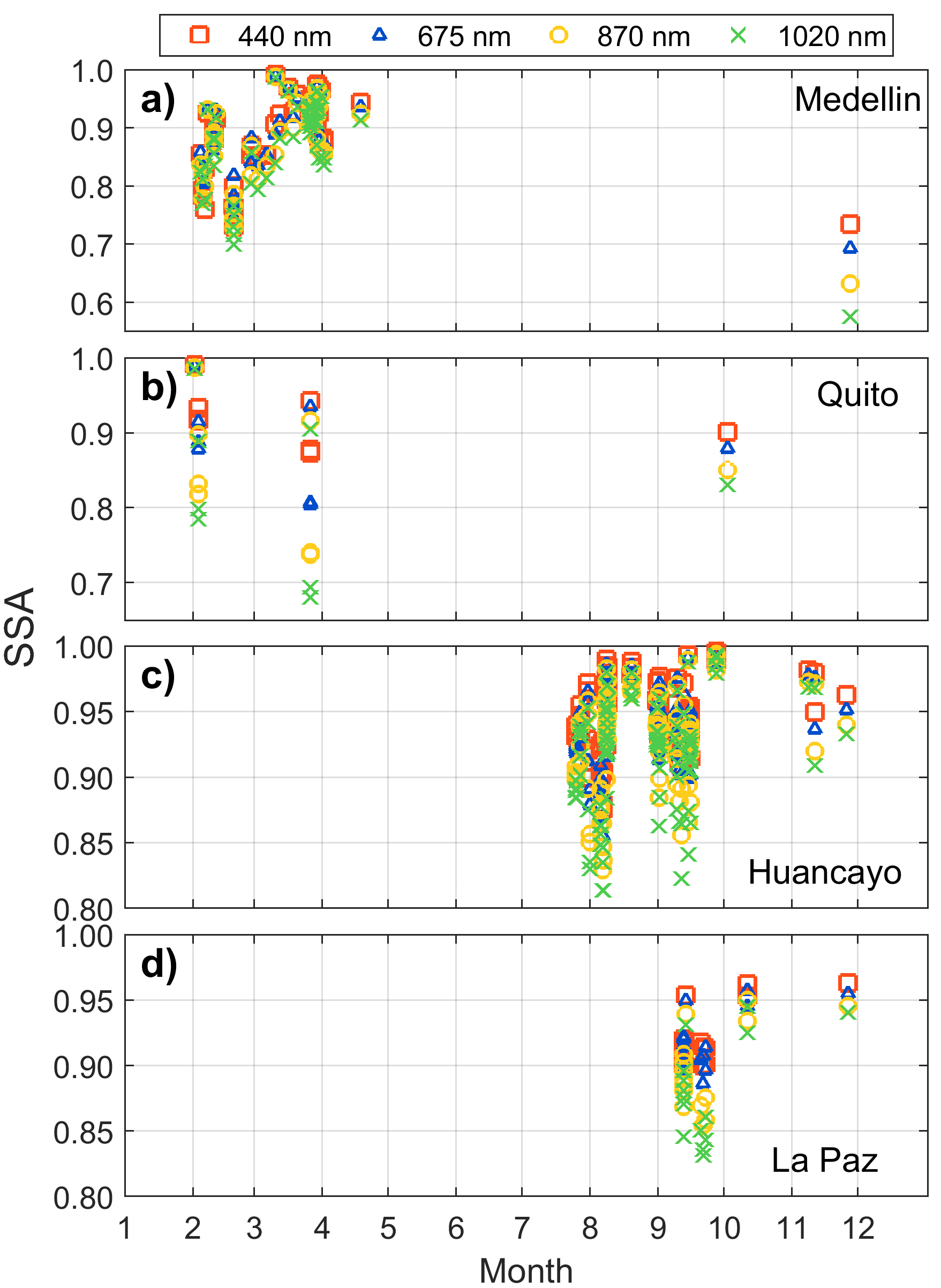


**Figures S6** AERONET Version 3 Level 2.0 SSA (single scattering albedo) events at 440, 675, 870, and 1020 nm from Hybrid and almucantar retrievals are overlapped in one year for a) Medellin, b) Quito, c) Huancayo, and d) La Paz.


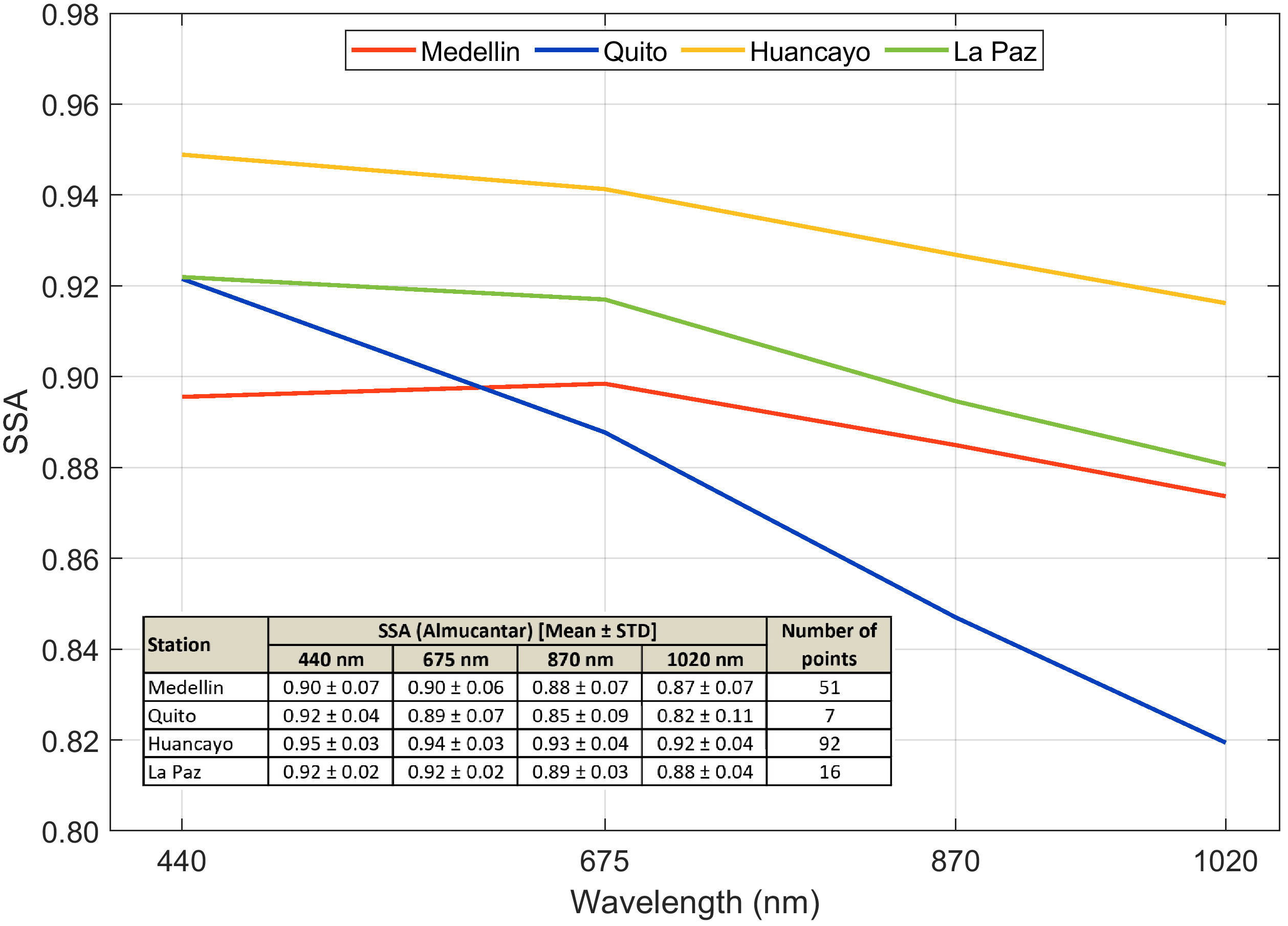


**Figure S7** SSA spectra (Hybrid and almucantar retrievals) from mean of observations plotted against wavelength for Medellin (red), Quito (blue), Huancayo (yellow), and La Paz (green) with statistics (mean +/- 1-standard deviation) inside table. At Medellin and Quito, the single event in November 2012 and October 2018, respectively, were excluded from the cluster analysis.


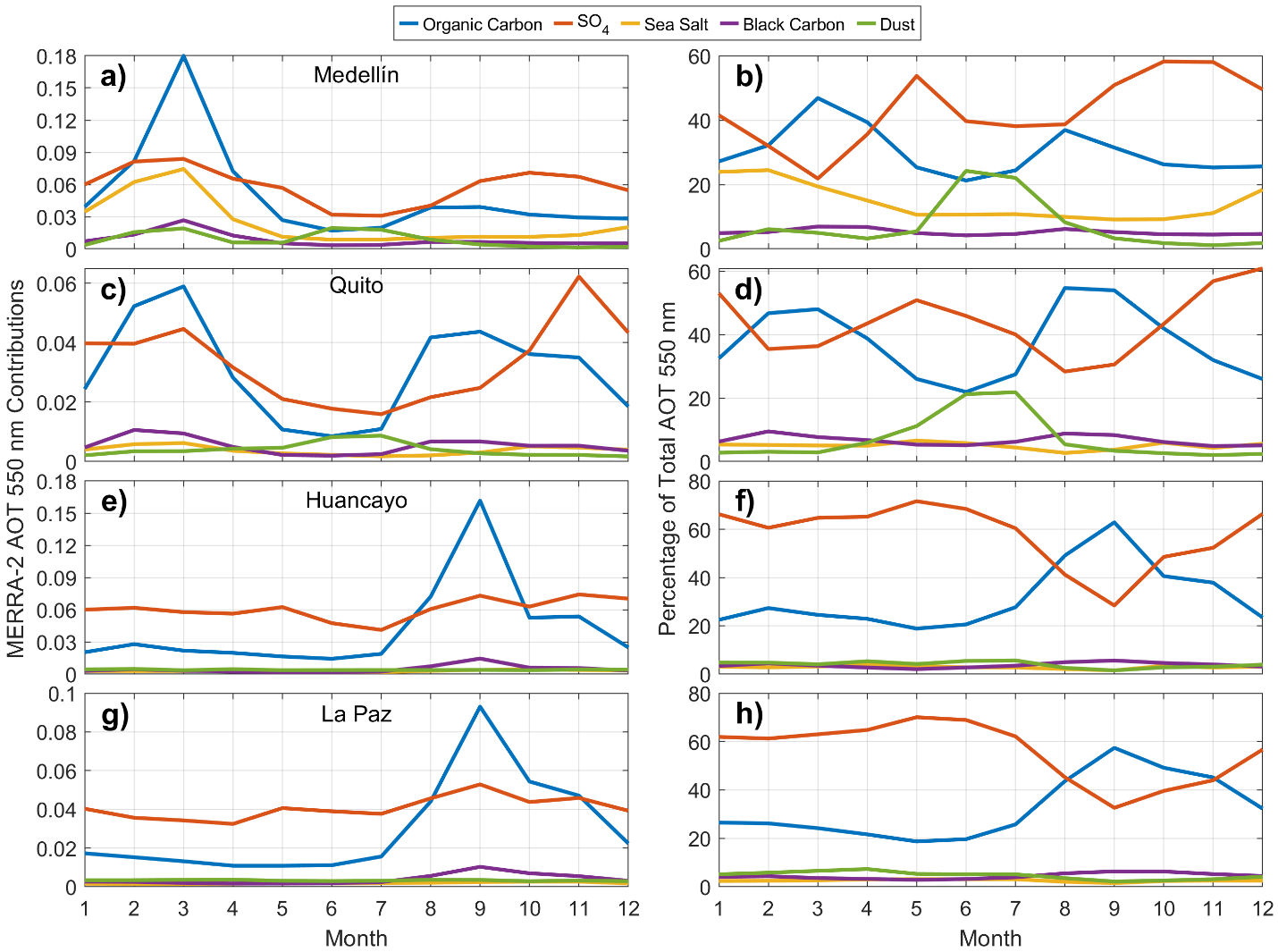


**Figures S8** MERRA-2 AOT 550 nm contributions due to organic carbon (blue), SO_4_ (red), sea salt (yellow), black carbon (purple), and dust (green) as annual cycles from monthly means (2006-2022) at Medellin, Quito, Huancayo, and La Paz. Left panels show AOT magnitude, while right panels show contributions as percentages.


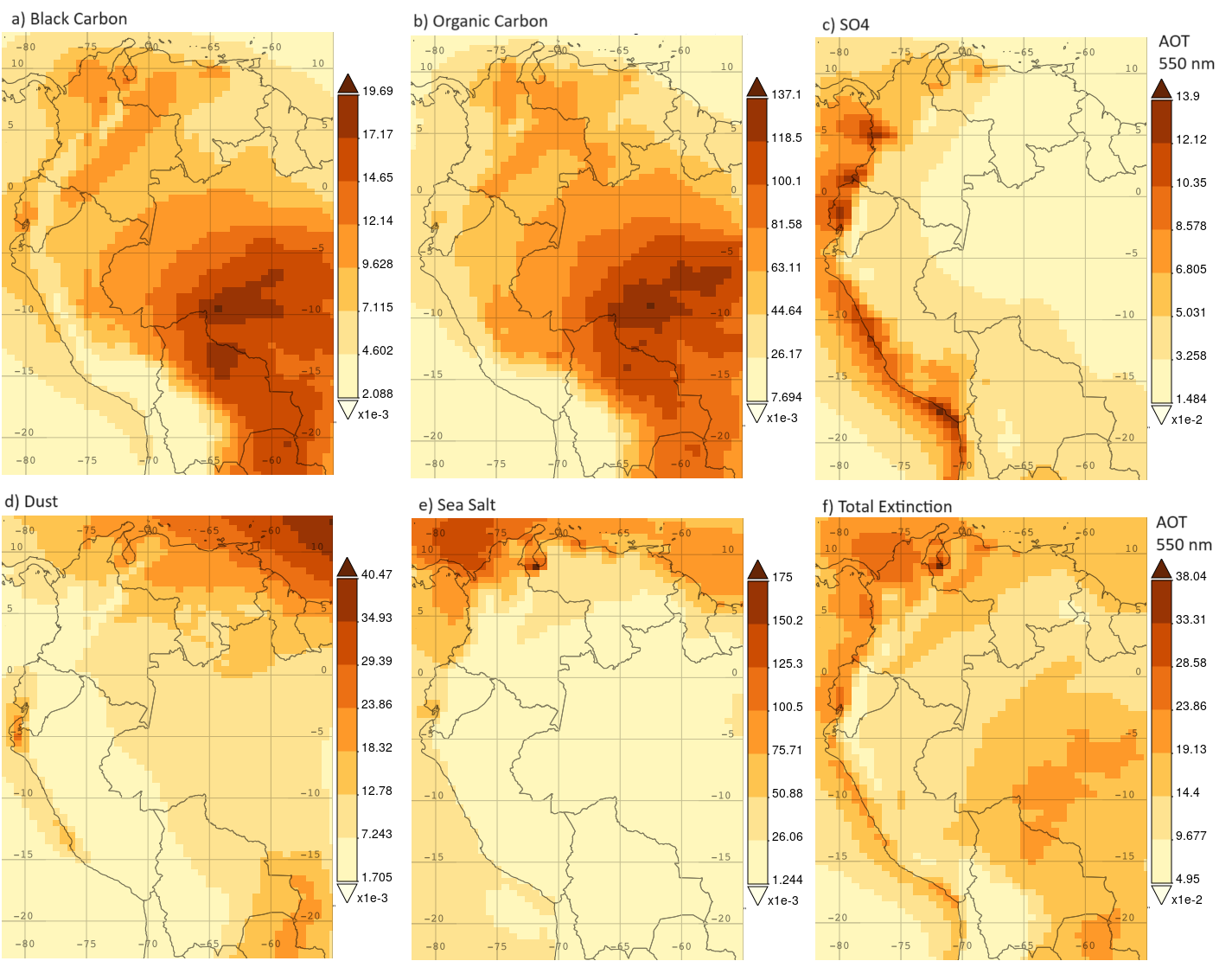


**Figure S9** 2012-2022 time-averaged maps of MERRA-2 extinction AOT 550 nm for a) black carbon, b) organic carbon, c) SO4, d) dust, e) sea salt, and f) total AOT. Maps were created online from the Giovanni NASA website (https://giovanni.gsfc.nasa.gov/giovanni/).
